# Quantifying Förster Resonance Energy Transfer from Single Perovskite Quantum Dots to Organic Dyes

**DOI:** 10.1021/acsnano.3c11359

**Published:** 2024-03-28

**Authors:** Leon G. Feld, Simon C. Boehme, Viktoriia Morad, Yesim Sahin, Christoph J. Kaul, Dmitry N. Dirin, Gabriele Rainò, Maksym V. Kovalenko

**Affiliations:** † Institute of Inorganic Chemistry, Department of Chemistry and Applied Biosciences, ETH Zürich, CH-8093 Zürich, Switzerland; ‡ Laboratory for Thin Films and Photovoltaics, Empa − Swiss Federal Laboratories for Materials Science and Technology, CH-8600 Dübendorf, Switzerland; § National Centre of Competence in Research (NCCR) Catalysis, ETH Zürich, CH-8093 Zürich, Switzerland

**Keywords:** perovskite, quantum dot, energy transfer, photocatalysis, photoluminescence, FRET

## Abstract

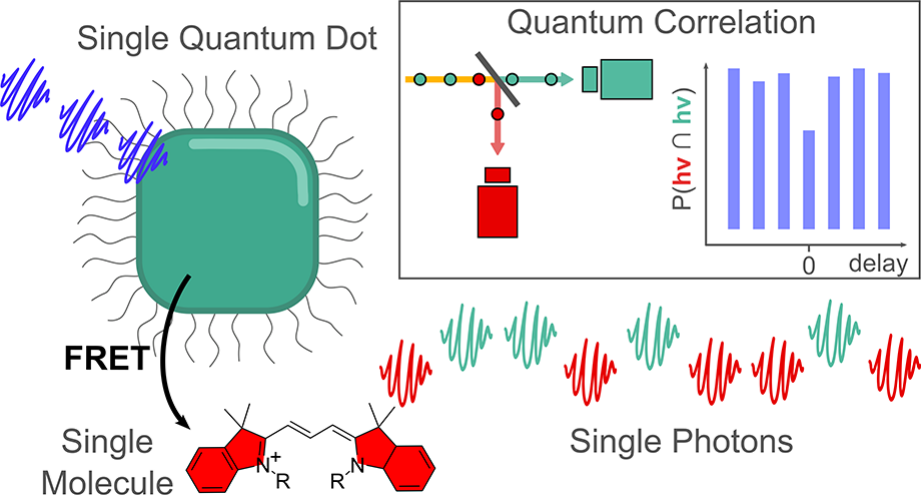

Colloidal quantum dots (QDs) are promising regenerable photoredox catalysts offering broadly tunable redox potentials along with high absorption coefficients. QDs have thus far been examined for various organic transformations, water splitting, and CO_2_ reduction. Vast opportunities emerge from coupling QDs with other homogeneous catalysts, such as transition metal complexes or organic dyes, into hybrid nanoassemblies exploiting energy transfer (ET), leveraging a large absorption cross-section of QDs and long-lived triplet states of cocatalysts. However, a thorough understanding and further engineering of the complex operational mechanisms of hybrid nanoassemblies require simultaneously controlling the surface chemistry of the QDs and probing dynamics at sufficient spatiotemporal resolution. Here, we probe the ET from single lead halide perovskite QDs, capped by alkylphospholipid ligands, to organic dye molecules employing single-particle photoluminescence spectroscopy with single-photon resolution. We identify a Förster-type ET by spatial, temporal, and photon–photon correlations in the QD and dye emission. Discrete quenching steps in the acceptor emission reveal stochastic photobleaching events of individual organic dyes, allowing a precise quantification of the transfer efficiency, which is >70% for QD–dye complexes with strong donor–acceptor spectral overlap. Our work explores the processes occurring at the QD/molecule interface and demonstrates the feasibility of sensitizing organic photocatalysts with QDs.

## Introduction

Optical properties and redox potentials of colloidal quantum dots (QDs) can be systematically tuned by altering the size and composition of their inorganic semiconductor core. Tunability is realized through a facile synthesis yielding colloidal dispersions of QDs that can be used as inks or separated from the solvent by precipitation. In addition, QDs feature extremely large extinction coefficients (>10^6^ M^–1^ cm^–1^)^1^ across a broad spectral range. These characteristics have thus far motivated applications of QDs in optoelectronics, particularly as classical^2^ and quantum^3^ light sources or infrared detectors.^4,5^ Colloidal QDs are also increasingly explored as photocatalysts^6−14^ owing to the broad absorption bands, large extinction coefficients and high photostability, the absence of a Marcus-inverted regime,^15−17^ and the facile separation of products from the photocatalyst by anti-solvent-induced precipitation.^6^

Lead halide perovskite QDs are the latest class of colloidal QDs, known for exhibiting near-unity photoluminescence (PL) quantum yields without the need of converting them into elaborate core–shell heterostructures.^18^ They can be produced at room temperature under atmospheric conditions from nonprecious metals in a scalable manner.^19^ While all perovskite QDs strongly absorb in the blue-UV region, fine control over the absorption onset and PL emission band across the entire visible spectrum can be achieved by tuning the halide composition or QD size during or postsynthesis.^18^ Lead halide perovskite compounds were reported to photocatalyze a variety of organic reactions including C–C couplings,^20−22^ N-heterocyclization, and C–O coupling.^23^ However, further advances in QD surface design are needed to enhance the stability and photocatalytic performance.^24−26^

**Figure 1:**
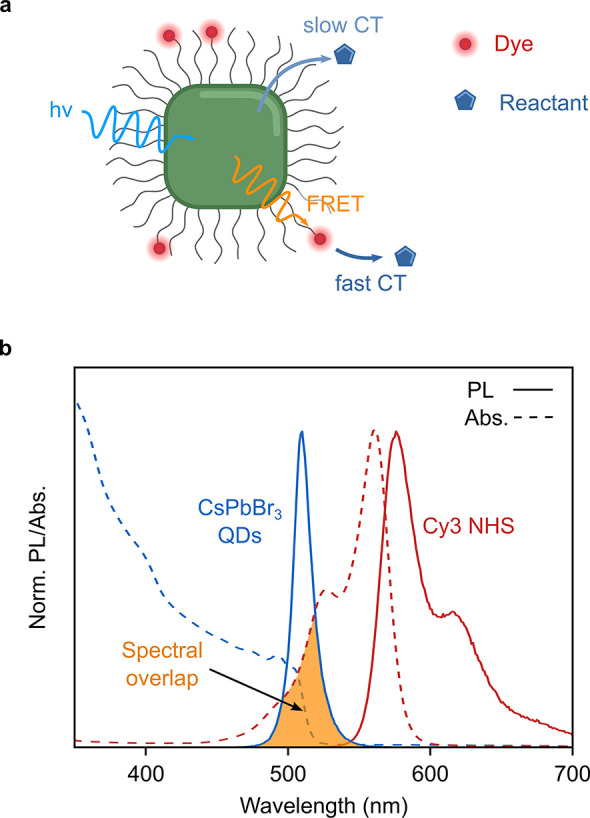
Perovskite QDs as photocatalysts. (a) Illustration of a QD with a nonpermeable ligand shell and its ET pathways to organic dyes. Direct charge/energy transfer to the reactant is slow (pale blue arrow), whereas the FRET to a dye (orange wave) and subsequent charge transfer (CT) to the reactant (blue arrow) can be faster. (b) Normalized PL and absorption spectra of the donor (CsPbBr_3_ QDs, blue) and acceptor (cyanine 3 NHS ester, red) used in this study. The yellow area indicates the spectral overlap of donor PL and acceptor absorption that is required for FRET.

Obtaining highly stable perovskite QD colloids requires robust capping ligands that ensure inorganic core integrity and favorable tail–solvent interactions for colloidal stabilization.^27−29^ However, in a photocatalytic context, a dense ligand shell with the thickness of one nanometer or more could prevent substrates from reaching the surface of the QDs at which charge and triplet energy transfer occur (Figure 1a).^11,25,30−33^ Thus, the photocatalytic activity relying on short-range energy/charge transfer will be impeded. On the other hand, Förster resonance energy transfer (FRET) could be a viable mechanism to transfer energy from QDs to organic molecules that cannot permeate the ligand shell. Since FRET is a long-range energy transfer (ET) mechanism via dipole–dipole coupling, i.e., via spectral overlap of donor PL and acceptor absorption, FRET occurs efficiently at donor–acceptor distances of up to ca. 10 nm.^34,35^

Whether FRET efficiently occurs between a perovskite QD and an organic dye is still an open scientific question due to the complex and heterogeneous nature of such hybrid systems. On one hand, the occurrence of FRET between perovskite donor QDs and CdSe has been suggested by ensemble-based PL studies, inferred from the peculiar dependence of ET efficiency on donor–acceptor distance.^26,36^ On the other hand, a profound contribution of short-range ET or charge transfer, both relying on overlap between donor and acceptor orbitals, was observed at very small donor–acceptor distances.^36,37^ In addition, a considerable deviation from the dependence on the spectral overlap predicted by FRET theory was found in the ET from perovskite QDs to organic dyes in ensemble PL and single-particle PL studies, suggesting a significant contribution by nonresonant ET.^38−40^ The latter could exclusively proceed through short-range ET, which requires orbital overlap established upon surface adsorption of ET acceptors. Isolating FRET as the sole ET mechanism requires the employing of a robust ligand shell that is not permeable to acceptor molecules.

The manifold of processes occurring in such composite nanoassemblies indicates their structural and electronic complexity. Disentangling the complex behavior arising at the QD–molecule interfaces requires resolving spatial inhomogeneities at the nanometer length scale and temporal fluctuations at short time scales, a persistent bottleneck even for the most advanced nanoscale characterization methods.^41^ In our work, we address this challenge by employing PL spectroscopy at the single-particle and single-photon levels. Optical single-particle techniques can resolve spatial heterogeneities^41,42^ and temporal fluctuations,^43−45^ which average out in the ensemble-level experiments. The method is therefore a valuable tool to study chemical reactions^46−48^ and to quantify nanoparticle–molecule interactions.^49,50^ Combining single-particle techniques with the distance dependence of FRET enables studying subpopulations, dynamics fluctuations, and rare events in heterogeneous and dynamic systems. Single-particle FRET thus became indispensable to study processes in biological systems.^51−53^

In this work, we perform single-particle PL studies employing CsPbBr_3_ QDs capped with 2-octyl-1-dodecylphosphoethanolamine (C_8_C_12_-PEA) ligands, which were found to strongly bind to the QD surface with a maximal ligand coverage.^29^ First, we find experimental evidence for the occurrence of FRET as the predominant ET mechanism, unlike in previous studies employing similar perovskite-QD–dye hybrids but without the recently developed nonpermeable ligand shell. Then, we determined the FRET efficiency between a single QD and single dye by counting the molecules that undergo FRET with the QDs as well as by analyzing the PL lifetime through time-resolved single-particle PL spectroscopy. Exploring several different molecules displaying varying spectral overlap with donor QDs corroborates our assignment of FRET as the transfer mechanism and highlights the importance of heterogeneity in the system response characteristics of molecular compounds. Our results explore the underlying mechanism responsible for the ET in perovskite-QD/organic-molecule composites featuring nonpermeable ligand shells and aid the design of more efficient photocatalysts. Toward this goal, we demonstrate efficient ET from perovskite QDs to perylene bisimide molecules, known to act as photoredox catalysts for reactions including C–H bromination, fluoroalkylation, or arylations.

## Results and Discussion

### Single-Particle FRET Samples

A recent work introduced a library of synthetic, zwitterionic phospholipid ligands for perovskite QDs with enhanced QD–ligand binding and stable dispersion in diverse media owing to the chemical tunability of the organic tail.^29^ For our model system in the present study, we opted for the C_8_C_12_-PEA ligand with a maximum stretched length of ca. 1.4 nm. C_8_C_12_-PEA-capped CsPbBr_3_ QDs of ca. 9.5(9) nm in size were prepared according to ref (29); see details in the Supporting Information, including Figure S1. Further processing of the samples was performed under inert conditions with dry apolar solvents. C_8_C_12_-PEA ligands afford significantly improved ligand passivation compared to other ligands, including other zwitterionic ligands.^29,54^ Single-particle FRET samples were prepared by mixing highly diluted CsPbBr_3_ QD dispersions and a solution of an organic dye, cyanine 3 NHS ester (Cy3), in significantly higher concentration in toluene and spin-coating the resulting solution onto cover glass substrates. This procedure yielded films with a sparse distribution of QDs (<1 QD/μm^2^) and a high density of Cy3 (see Supporting Information for details on the film preparation). Since Cy3 absorption affords a good spectral overlap with the CsPbBr_3_ QD emission, FRET is expected at sufficiently short donor–acceptor distances (see Figure 1b).

### Spatial, Temporal, and Photon–Photon Correlation

**Figure 2:**
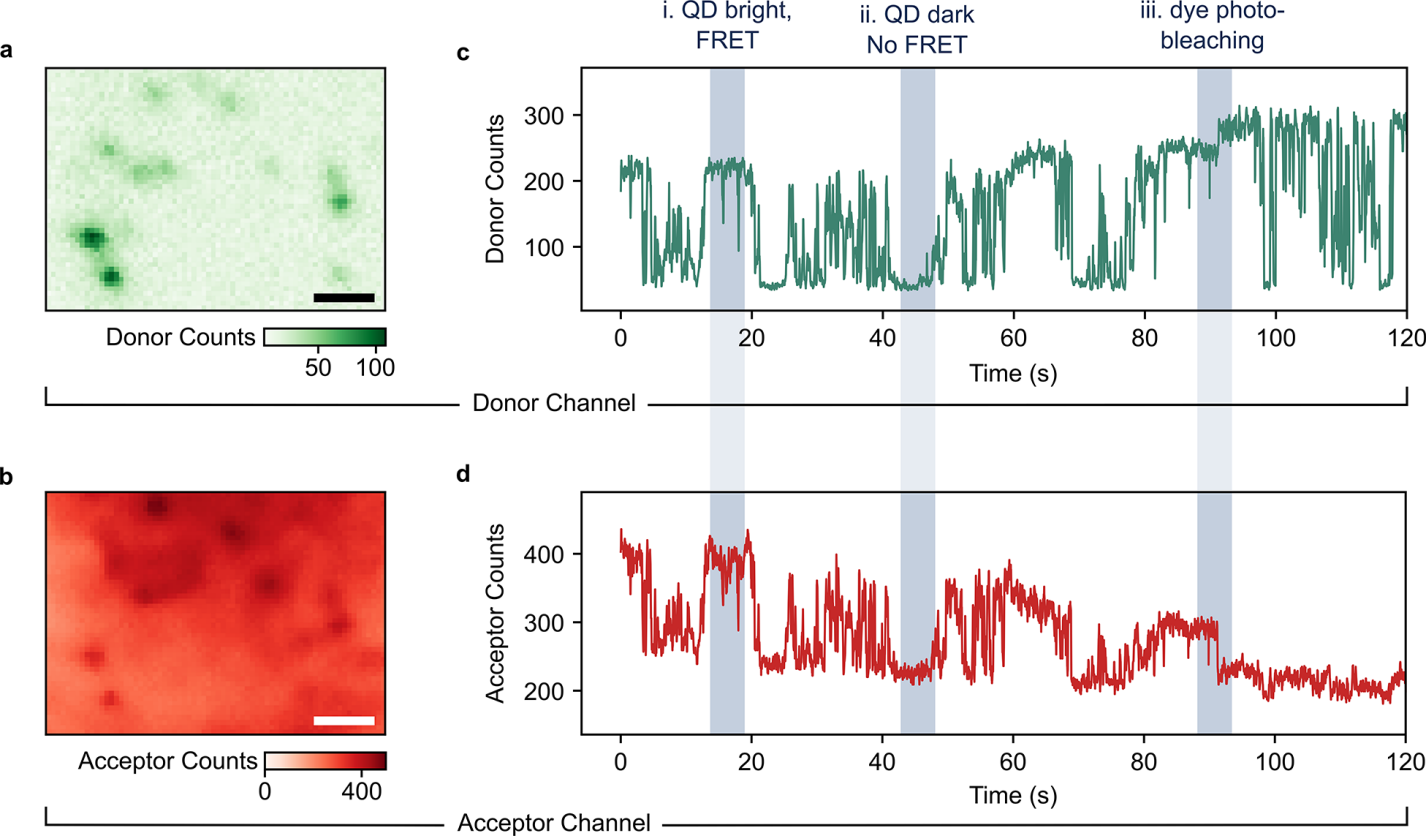
Spatial and temporal donor–acceptor correlations. (a, b) Wide-field images selectively probing the green emission of QDs (a) and the red emission from dyes (b) show a spatial correlation of bright spots in the two channels. Scale bars correspond to 2 μm. (c, d) Time traces (10 ms time-binning) of the green QD donor (c) and red dye acceptor (d) emission in a bright spot demonstrating strong temporal correlation. Three distinct types of correlation are highlighted via gray bars, namely, (i) the presence of FRET if the QD is in a bright state, (ii) the absence of FRET if the QD is dark, and (iii) photobleaching events of the dye leading to an increase in the QD emission. Note: Vertical axes do not include zero counts.

To observe and probe ET, we first study the spatial correlation of QD and dye emission via wide-field imaging in a home-built single-particle fluorescence microscope upon excitation with a 405 nm pulsed laser (details in SI). Optical properties of bare single QDs are reported in Figure S4. Figure 2a displays wide-field images, selectively probing either only green QD PL or red dye PL, obtained by passing the emitted light through a band-pass filter (510 nm central wavelength, 42 nm bandwidth) or a long-pass filter (550 nm cut-on), respectively. In the green QD channel, we observe individual bright spots corresponding to emission from the spatially well-separated single QDs. Second-order correlation measurements clearly exhibit antibunching behavior, attesting to the single-photon emission properties of isolated QDs (Figure S5). In the red dye channel, we observe a constant background emission with few localized bright spots. The constant background arises from direct excitation of a dense layer of Cy3, with finite absorption at 405 nm (Figure 1b), observed also in a reference sample without QDs but not in the absence of the dye (Figure S6). The few bright spots feature a high spatial correlation with the bright spots in the green QD channel, suggesting that QDs undergo ET to dye molecules and thereby act as an antenna for the dye molecules.^40^

Next, the temporal correlation between the QD donor PL and the dye acceptor PL was studied by focusing the excitation beam on individual QDs and recording the emitted light in a spectrally sensitive Hanbury-Brown–Twiss (HBT) experiment (Figure S7). One of the two HBT arms records the green QD donor PL, utilizing the above-described bandpass filter, while the other arm simultaneously records the red dye acceptor PL, utilizing a long-pass filter with cut-on wavelength at 550 nm. Figure 2c shows an intensity time trace from the QD donor with intensity fluctuations resembling the blinking behavior typically observed in perovskite QDs and other nanoscale emitters as they switch between optically bright and dark states.^43,44,55−58^ The trace in the dye acceptor channel (Figure 2d) exhibits intensity fluctuations closely correlated with QD donor blinking. Particularly, we observe three types of donor–acceptor correlations that are highlighted in the time traces (Figure 2c and d): (i) if the QD is in its bright state, there is bright emission in the red dye channel, (ii) if the QD is in its dark state, there is no emission in the red dye channel besides the background, and (iii) a step-like decrease in the red dye channel is accompanied by a step-like increase in the green QD channel. These three scenarios correspond to (i) the presence of ET if the QD is bright, (ii) the absence of ET if the QD is dark, and (iii) photobleaching of a dye acceptor molecule and thereby less quenching of the QD donor emission.

**Figure 3:**
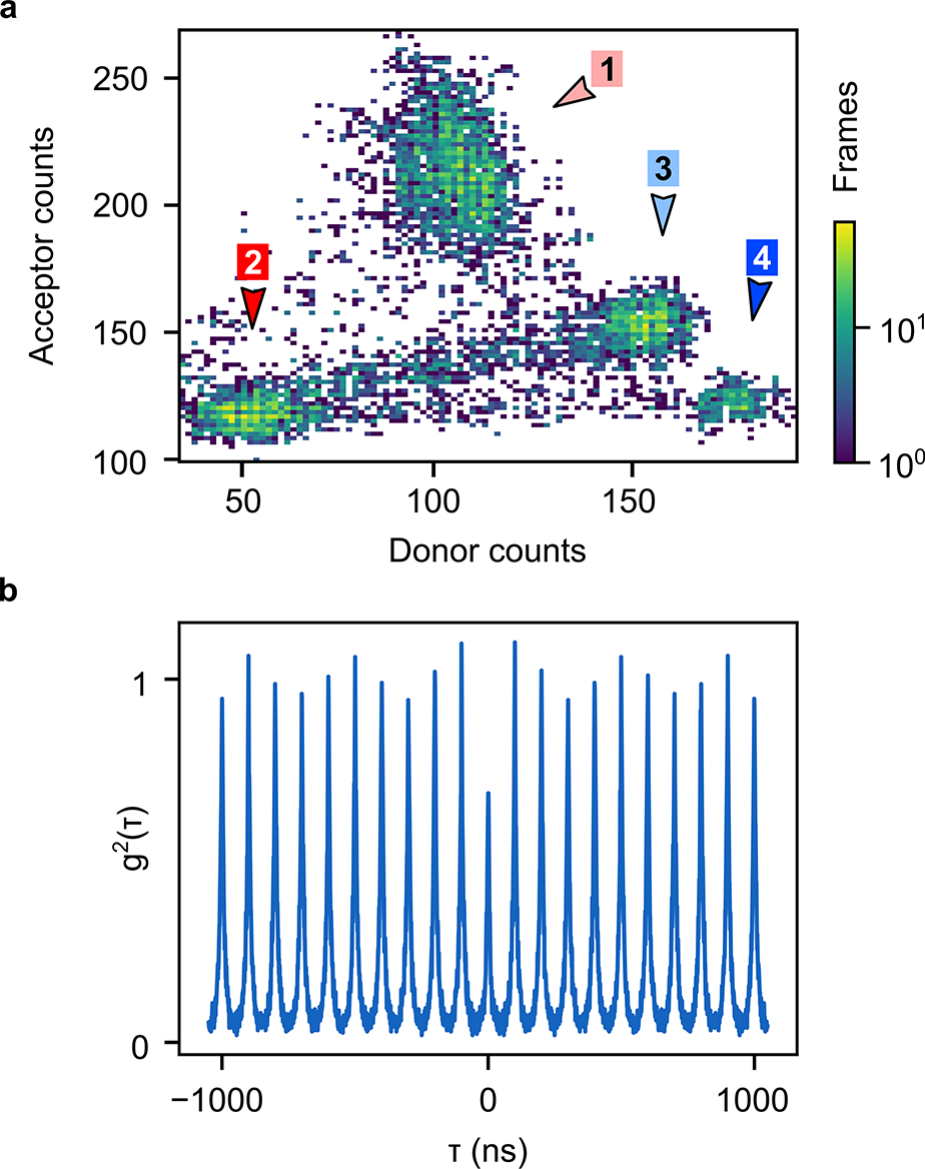
Intensity and photon–photon correlations of the QD and the Cy3 emission. (a) Correlation of the intensities (10 ms time-binning) in the green QD donor and the red dye acceptor channel. The probability is indicated by a logarithmic heatmap, and the types of correlation are indicated by arrows: bright QD and bright dyes due to ET (light red, 1), dark QD and dark dyes due to quenched QD emission with the absence of ET (red, 2), brighter QD and darker dyes after photobleaching of the first (light blue, 3) and second dye (dark blue, 4). Note: Axes do not include zero counts. (b) Second-order correlation function (*g*^2^(τ)) of the donor and acceptor photon arrival times. The antibunching (dip at a zero-delay time) indicates anticorrelated emission of donor and acceptor.

Figure 3a further quantifies the intensity correlations between the red dye acceptor channel and the green QD donor channel via a two-dimensional correlation map obtained from intensity time traces similar to those in Figure 2c,d. Again, three distinct types of correlation emerge: (i) a cluster at the highest dye intensity and high QD intensity (light red arrow) is assigned to a bright QD undergoing ET to two dye molecules; (ii) a cluster at both low dye and low QD intensity (red arrow) corresponds to the absence of ET when the QD is found in its dark state; (iii) once the first dye is photobleached, the intensity decreases in the red dye acceptor channel and increases in the green QD donor channel (light blue arrow), consistent with decreased ET due to loss of one of the acceptors; finally, after also the second dye undergoes photobleaching, a cluster emerges with an even brighter QD and negligible acceptor intensity, consistent with no ET due to the lack of suitable acceptors (blue arrow).

Although we have already presented nanometer-scale spatial correlation in the images and millisecond temporal correlation in the intensity time traces and correlation map, we now provide unambiguous proof for assigning these observations to ET between the QD and the dye via photon–photon correlations on a picosecond time scale. To this end, we search for anticorrelation of the arrival times of individual photons in the two spectral channels of the HBT experiment.^59,60^ Upon creation of a single exciton in a single QD by the laser pulse, the exciton in the QD can either radiatively decay in the QD, emitting a green photon, or be transferred to a molecule via ET with subsequent emission of a red photon from the dye, but not via both decay channels simultaneously. Therefore, both red and green photons should not be observed following the same excitation pulse, and hence, the signals of the two HBT arms should be anticorrelated. In the ideal case, the associated photon–photon correlation function *g*^2^(τ) would be fully “antibunched”, i.e., *g*^2^(0) = 0. In our case, Figure 3b shows that the *g*^2^(0) belonging to one of the bright spots in the wide-field images still shows a finite value due to the presence of the uncorrelated background in the red acceptor channel via direct laser excitation of the dye (see above). The total measured *g*^2^(τ) is the sum of the correlation of photons emitted by the QD with photons emitted by dye molecules after indirect excitation via ET (*g*^2^(0) ≪ 1) and with photons emitted by the dye molecules after direct laser excitation (*g*^2^(0) = 1). Thus, the pronounced dip at τ = 0 (*g*^2^(τ) < 1), discernible even in the likely presence of directly excited dyes, confirms that QDs and dyes are quantum-mechanically coupled.

### Energy Transfer to Nile Red

After demonstrating ET from perovskite QDs to Cy3 dyes at the single-particle level, we extend our studies to the dye Nile Red to test the generality of the proposed FRET mechanism. Since Nile Red features good spectral overlap of its absorption with the QD emission (Figure S8), a response similar to that for Cy3 is anticipated. However, other photophysical properties, such as PL quantum yield, blinking, and photostability, are specific to the employed molecular acceptor system and, hence, may alter the experimental observations. In fact, while both the PL and absorption of Cy3 in solution (Figure 1) and film (Figure S9) do not differ significantly, the PL of Nile Red is fully quenched in a dense film (Figure S9). Consequently, unlike in Cy3, the red emission of the Nile Red molecules excited by ET from the QDs strongly fluctuates with only short bursts of PL (see Figure S10). Nevertheless, similarly as for Cy3, we find pronounced QD–dye spatial correlations in wide-field images (Figure S11) as well as QD–dye temporal correlations in intensity time traces (Figure S10), correlation map, and *g*^2^(τ) (Figure S12). The qualitatively similar spatial and temporal correlations in the ET transfer for Nile Red and Cy3 show that the ET can be generalized to different dyes. Furthermore, a single-particle ET monitoring allows us to recover characteristics of the acceptor dyes such as strong blinking in the case of Nile Red molecules.^61^

### Dependence on Spectral Overlap

Previous studies on similar perovskite-QD–dye systems reported mechanisms different from FRET, i.e., nonresonant short-range ET.^38,39^ To clarify the discussion and confidently assign our here-observed ET mechanism to FRET, we therefore test whether (i) ET requires spectral overlap of donor emission and acceptor absorption and (ii) the ET efficiency exhibits the FRET-characteristic donor–acceptor distance dependence, *vide infra*. To verify requirement (i), we replace Cy3 in our experiments with cyanine 5 NHS ester (Cy5) and thereby eliminate the spectral overlap between the QD emission and dye absorption by an order of magnitude (Figure S13; details in SI). Unlike for the dyes with strong spectral overlap, neither spatial correlation in wide-field images (Figure S14) nor temporal correlations in intensity time traces (Figure S15), correlation maps, or *g*^2^(τ) (Figure S16) can be observed. Hence there are no signs of nonresonant ET resulting in acceptor emission. We further study the PL lifetime of the single QDs in the presence of Cy5 by performing time-resolved PL measurements. Unlike for the QDs with Cy3 (*vide infra*), the PL lifetime remains unchanged (Figure S17). The dependence on spectral overlap is also observed in ensemble-level time-resolved PL measurements of QDs in the presence of Cy3 or Cy5 (Figure S18).

Alternatively, the spectral overlap of QD PL and dye absorption can also be reduced by an order of magnitude by pairing Cy3 with mixed halide CsPb(Br/Cl)_3_ QDs exhibiting an ensemble PL centered at around 465 nm (Figure S19; details in SI). Again, we observe no signs of correlation (Figures S20). Yet another route toward decreasing the spectral overlap is to decrease the donor and acceptor spectral line widths via freezing out vibrational modes through cooling to cryogenic temperature. Employing this method, a previous study^39^ demonstrates nonresonant ET between perovskite QDs and Cy3 at cryogenic temperature. Different from this previous study, our QD-Cy3 sample with the nonpermeable ligand shell, previously displaying resonant ET at room temperature, predominantly does not exhibit nonresonant ET; only a minority of single QDs displayed ET to Cy3 (Figure S21). The entirety of these observations supports the assignment of FRET as the dominating ET mechanism in the QD–Cy3 and QD–Nile-Red systems, while the observation of nonresonant ET in some cases highlights the complexity and inhomogeneity in QD–dye interactions.

Nonresonant ET of Dexter-type would occur at short-range and is based on direct QD–dye contact that requires a permeable ligand shell. Dye molecules are likely surface-adsorbed in experiments that employ perovskite QDs with more labile (easy-to-desorb) alkylammonium coating and show nonresonant Dexter-type ET.^38,39^ On the other hand, our observation of FRET indicates that C_8_C_12_-PEA ligands provide a dense surface coating that largely prevents dye molecules from permeating the ligand shell. For QDs with labile ligands, we have recently found that the electronic passivation of the QD surface is degraded at the high dilution levels required to prepare single-particle samples.^29,54^ In contrast, the stronger binding of C_8_C_12_-PEA ligands to the surface of perovskite QDs yields a better surface passivation and a significantly higher resilience to dilution due to a much reduced ligand desorption.^29,54^ Nevertheless, sporadic observations of nonresonant ET in single-QD samples at cryogenic temperatures indicate that the permeability of dyes to the QD surface remains finite even for this latest ligand class.

### Quantification of FRET Efficiency

**Figure 4:**
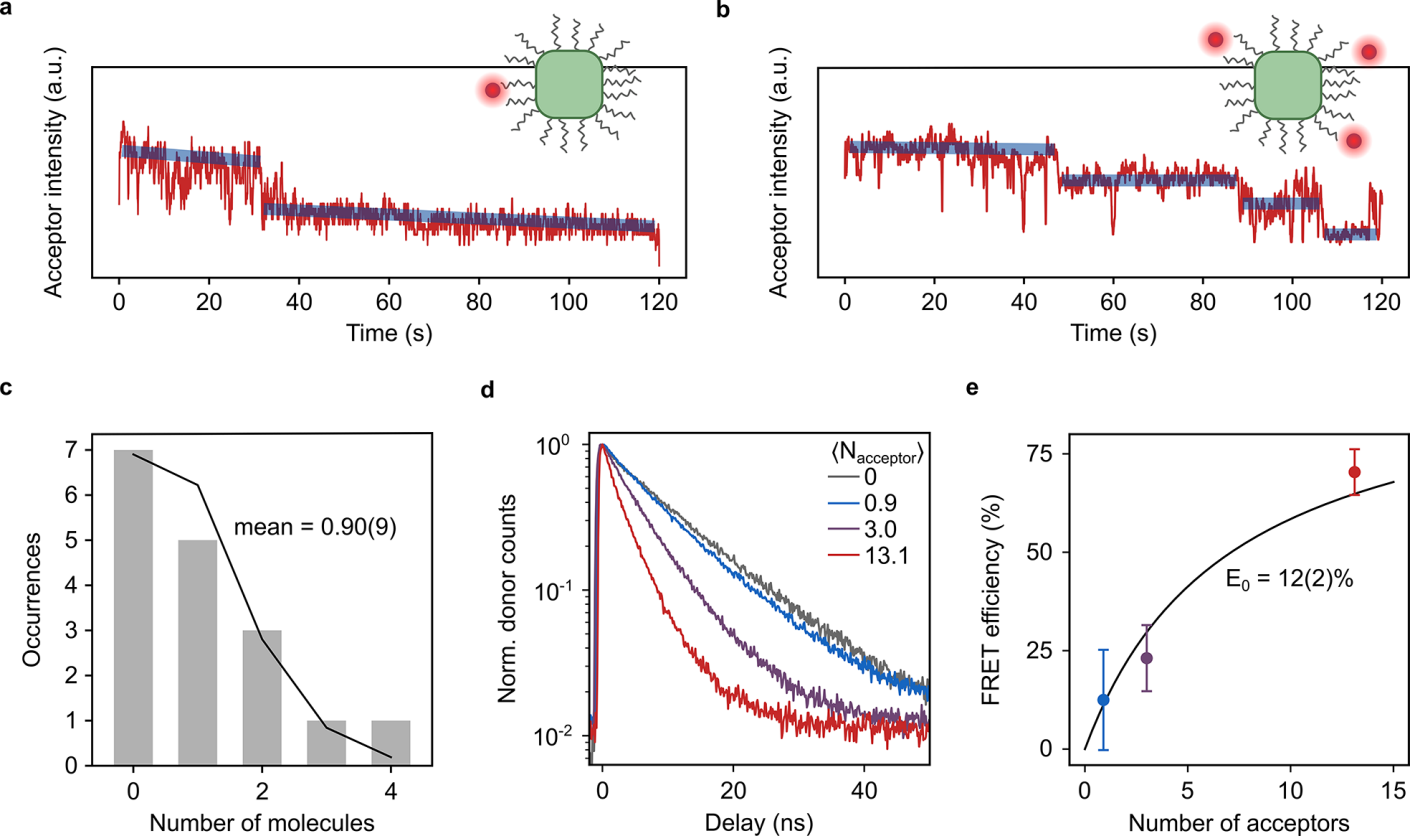
Number of acceptors and FRET efficiency for a single QD. (a, b) Intensity in the red acceptor channel for a single QD with one (a) and three molecules (b) undergoing FRET with the QD. The transparent blue lines serve as a guide to the eye and indicate the stepwise photobleaching of the molecules. Note that vertical axes do not start at zero counts. (c) Histogram of the counted acceptor molecules per single QD (gray bars). The black line corresponds to a fitted Poisson distribution with a mean number of molecules of 0.90(9). (d) Representative time-resolved PL traces of single QDs in samples without dye (gray line) and with increasing amounts of dye (blue to red lines). (e) Dependence of the sample-averaged single-particle FRET efficiency on the number of accepting molecules obtained from single-particle QD excited-state lifetimes estimated as 1/*e* decay times. The black line corresponds to a fit of eq 1 to the data, yielding a single-QD-to-single-dye efficiency *E*_0_ = 0.12(2), and error bars correspond to 95% confidence intervals.

Better comprehension of the nature and efficiency of QD–dye coupling through FRET requires knowledge of the composition of the nanoassembly. We determine the number of acceptor molecules surrounding the QD as the number of step-like photobleaching events in the intensity time trace of the acceptor,^62−65^ adopting an approach from single-molecule fluorescence studies.^66,67^Figure 4a and b show two examples of intensity time traces in the acceptor channel for a sample with a relatively low dye concentration. Here, QDs undergo efficient FRET only for a small number of accepting dye molecules, facilitating the determination of the number of acceptor molecules. Observing one and three bleaching steps before reaching the background level, we deduce that one and three accepting dye molecules were involved in the ET process, respectively. Figure 4c reports the statistics over several spots, with a fit to a Poisson distribution yielding a mean number of 0.90(9) dye per QD. Using this value, the number of accepting molecules for higher dye concentrations was estimated by assuming a linear dependence of the dye loading per QD and the dye concentration in the solutions used for spin-coating.

Next, we assess the FRET efficiency *E*_0_ between a single QD and a single dye molecule. Assuming identical transfer rates to each acceptor, the overall FRET efficiency for a QD interacting with *n* surrounding dye acceptor molecules is^68^
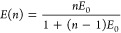
1While our single-particle level experiments suggest considerable variations within and across the nanoassemblies (see also the error bars in Figure S22), the assumption of identical transfer rates and the hereby obtained simple expression in eq 1 allow a straightforward estimate of the single-donor–single-acceptor FRET efficiency *E*_0_ once the overall efficiency *E*(*n*) and *n* are known. *E*(*n*) can be obtained from the excited-state lifetimes employing time-resolved PL measurements at the single-particle level:^68^
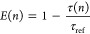
2where τ(*n*) and *τ*_ref_ are the QD lifetimes in the presence and absence of *n* accepting dye molecules per QD, respectively.

Representative single-particle time-resolved PL traces for the reference sample and three different dye concentrations are shown in Figure 4d, with a clear shortening of the lifetime with increasing acceptor concentration, also observed in ensemble experiments (Figure S23). In agreement with previous works,^69^ single-particle time-resolved PL measurements uncover large inhomogeneities in the excited-state lifetimes in both the absence and presence of dye molecules (Figure S22). Inhomogeneities in the absence of dye are caused by QD-to-QD variations of size^70^ and quantum yield or by blinking.^71,72^ In the presence of dye, they could be additionally caused by variations in ET rates through inhomogeneities in the number of acceptors (Figure 4c) and donor–acceptor distance.^73^

Figure 4e displays the sample-averaged FRET efficiency, obtained via eq 2 as a function of the estimated number of acceptor molecules per QD (details in the Supporting Information). The data points are well fitted by eq 1, yielding a FRET efficiency for a single QD–dye pair of *E*_0_ = 12(2)%. Already at <15 molecules per QD, the overall FRET efficiency reaches 70%, comparable to what was previously observed in an ensemble film with a high acceptor-to-QD ratio^26^ or with a large amount of surface-adsorbed dye molecules.^38^ The ability of FRET to compete with radiative recombination illustrates its fast kinetics. A similar dependence of the overall FRET efficiency on the acceptor concentration was observed also for Nile Red (Figure S24).

### Distance Dependence of FRET

While classical FRET formulations treat donor and acceptor as point dipoles, the Wannier–Mott excitons in perovskite QDs are extended objects, and their ET may thus require a multipole treatment. To provide clues whether or not our large perovskite QDs can still be approximated as point dipoles, we assess the universally used donor–acceptor distance dependence in such dipole-based FRET formulations:^68^
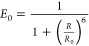
3where *R* is the donor–acceptor distance and *R*_0_ is the Förster radius, defined as the distance at which the efficiency is 50%.^68^ For the pairs of CsPbBr_3_ QD and Cy3, we obtain a Förster radius of 4.59 nm (details in SI), which is comparable to CdSe-QD–Cy3 pairs.^74^ From the value for *R*_0_ and *E*_0_ = 0.12(2), eq 3 then derives a donor–acceptor distance of *R* = 6.40(20) nm. This value is close to the minimum distance between the QD center and the outer side of the ligand shell of 5.8(4) nm, inferred from transition electron microscopy (TEM) images (Figure S3). Hence, the donor–acceptor distance responsible for FRET coarsely coincides with the distance between the dye and the QD center, i.e., the location where the donor exciton has its highest probability density.^75^ Overall, the agreement of TEM- and FRET-based distance estimation may both indicate the validity of the dipole approximation and strengthen our assignment of the QD–Cy3 ET mechanism to FRET.

### Sensitizing Photocatalysts

**Figure 5:**
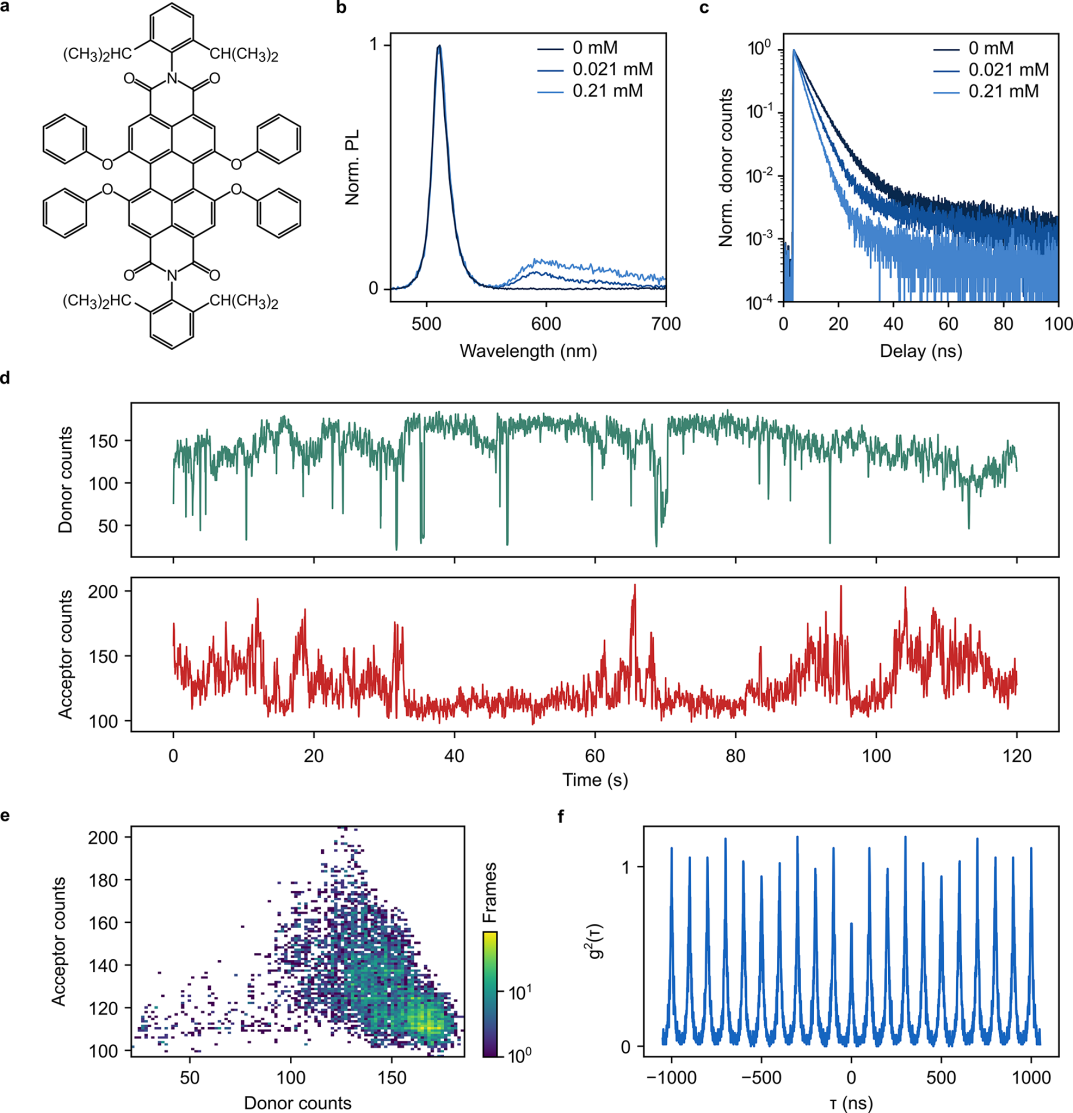
Energy transfer from CsPbBr_3_ QDs to a perylene bisimide (PBI) dye. (a) Structural formula of the PBI dye *N*,*N*′-bis(2,6-diisopropylphenyl)-1,6,7,12-tetraphenoxy-3,4,9,10-perylenetetracarboxylic diimide. (b) Ensemble PL spectra of CsPbBr_3_ QDs at increasing dye concentrations. (c) Time-resolved PL of the QD donor at increasing dye concentrations. (d) Time traces (10 ms binning) of green donor (top) and red acceptor (bottom) intensity in a bright spot of a single QD. (e) Intensity correlation map corresponding to the traces in (d), displaying anticorrelation of donor and acceptor counts. (f) Second-order correlation function (*g*^2^(τ)) of green donor and red acceptor photons exhibiting antibunching.

The hypothesis that FRET could be a viable mechanism to sensitize photocatalysts has so far only been tested with dyes that are photochemically inactive. We now investigate ET to a perylene bisimide (PBI)-based photocatalyst (Figure 5a).^76^ PBI molecules can act as photoredox catalysts for reactions including C–H bromination, fluoroalkylation, or arylations.^77,78^Figure 5b displays ensemble PL spectra of CsPbBr_3_ QDs in the presence of increasing concentrations of the PBI dye. The PL peak above 550 nm corresponds to the emission from the PBI dye^79,80^ which is sensitized by the QD, as identified by the shortened donor lifetime in time-resolved PL measurements (Figure 5c). At the single-particle level, intensity time traces of the green donor and red acceptor emission show (anti)correlated behavior signaling the occurrence of ET (Figure 5d). Moreover, intensity and photon–photon correlations corroborate this observation (Figure 5e,f).

These observations of efficient ET to organic photocatalysts across a highly insulating ligand shell demonstrate the feasibility of photocatalysts based on organic photocatalysts strongly sensitized by QDs. Ideally, photocatalysts would be tethered to the sensitizer via ligands to form colloidally stable nanoassemblies with fast energy funneling to the reaction site.

## Conclusion

We have characterized ET between single CsPbBr_3_ perovskite QDs and a small number of adjacent Cy3 molecules based on spatial, temporal, and photon–photon correlations of the QD and dye emission. The ET is assigned to FRET due to its characteristic dependence on both the spectral overlap and the distance between the donor and acceptor. Using single-particle spectroscopy, we resolved the stoichiometry of QD–dye assemblies, allowing us to deduce a FRET efficiency of 12% from a single QD to a single dye molecule. For higher dye loadings, the overall FRET efficiency from a single QD reaches 70% for only 15 acceptor molecules. Combined with the QDs’ large absorption cross-section, the FRET-based antenna effect enhances the effective dye absorption by 2 orders of magnitude. To realize efficient FRET in QD–dye assemblies and prevent charge transfer, previous limitations in surface passivation of perovskite QDs were overcome by utilizing a recently developed synthesis with enhanced ligand passivation and, hence, improved QD surface ligand coverage. Efficient FRET was also realized between CsPbBr_3_ QDs and an organic photocatalyst and could in the future be extended to deterministically formed nanoassemblies of QDs and organic photocatalysts, which could provide a viable route for photocatalyst designs.

## Methods

### Colloidal QD Synthesis

#### PbBr_2_-Trioctylphosphine Oxide (TOPO) Stock Solution (0.067 M)

PbBr_2_ (734 mg, 2.00 mmol, 99.999% trace metals basis, Sigma-Aldrich, stored in GB) and TOPO (4296 mg, 11.11 mmol, TOPO, 99%, Strem, stored in GB) were dissolved in 25 mL of *n*-octane (for synthesis ≥99%, Carl Roth) at 120 °C on a hot plate in air.

#### ZnCl_2_-TOPO Stock Solution (0.067 M)

ZnCl_2_ (273 mg, 2.04 mmol) and TOPO (4296 mg, 11.11 mmol) were dissolved in *n*-octane (25 mL) at 120 °C on a hot plate in air.

#### Cs-Diisooctylphosphinic Acid (DOPA) Stock Solution (0.02 M)

Cs_2_CO_3_ (97.8 mg, 0.30 mmol, 99.9% trace metals basis, Sigma-Aldrich) and DOPA (1 mL, 3.15 mmol, technical ≈90%, Sigma-Aldrich) were dissolved in 2 mL of *n*-octane (for synthesis ≥99%, Carl Roth) at 120 °C on a hot plate in air. The reaction mixture was allowed to cool to room temperature, then diluted with 27 mL of *n*-hexane (suitable for HPLC ≥ 97.0%, Sigma-Aldrich, stored over molecular sieves).

Ligand 2-octyl-1-dodecyl phosphoethanolamine (C_8_C_12_-PEA) was synthesized according to ref (29).

#### CsPbBr_3_ QD Synthesis

PbBr_2_-TOPO (260 μL) was diluted with *n*-hexane (1 mL) and stirred on a stirring plate. CsDOPA (300 μL) was swiftly injected. QDs were allowed to grow for 60 s, and then 2 mg of C_8_C_12_-PEA in 20 μL of mesitylene was injected to stop the QDs’ growth. For purification, QDs were precipitated with 1 equiv of an EtOAc–ACN (2:1 v/v) mixture; the precipitate was collected and redispersed in *n*-hexane. The purification procedure was repeated twice, and in the final step the QDs were redispersed in *n*-octane.

#### CsPb(Br/Cl)_3_ QD Synthesis

PbBr_2_-TOPO (260 μL) and ZnCl_2_-TOPO (100 μL) were diluted with *n*-hexane (1 mL) and stirred on a stirring plate. CsDOPA (300 μL) was swiftly injected. QDs were allowed to grow for 5 min, and then 2 mg of C_8_C_12_-PEA in 20 μL of mesitylene was injected to stop the QDs’ growth. The purification procedure followed that of the CsPbBr_3_ sample above.

### Transmission Electron Microscopy

TEM images were collected using a Hitachi HT7700 microscope equipped with a tungsten/LaB_6_ emitter and a double-gap objective lens system, operated at 100 kV. TEM images were processed using the software ImageJ.

### Ensemble Optical Characterization

The UV–vis measurements were conducted with a V670 spectrometer from Jasco, equipped with a photomultiplier tube (PMT) and a Peltier-cooled PbS detector in transmission mode. The transmission spectra were corrected for a dark-count spectrum and referenced to a baseline spectrum of the employed solvent.

The PL spectra were recorded with a Fluorolog iHR 320 Horiba Jobin Yvon spectrometer from Horiba Scientific fitted with a PMT detector. The PL emission was determined by finding the emission wavelength λ_emission_ with a maximum PL intensity.

Time-resolved PL measurements were performed with a FluoTime 300 spectrometer from PicoQuant equipped with a TimeHarp 260 PICO counting TCSPC unit and a 355 nm pulsed PicoQuant laser. Samples were prepared by spin-coating or drop-casting of solutions containing QDs (∼0.1 mg/mL) and organic dyes (concentrations indicated in figure legends). Donor decay traces were recorded at the PL peak center by using an emission monochromator.

### Single-Particle Spectroscopy

Sample preparation was performed in a glovebox that is kept under a nitrogen atmosphere, employing dry and filtered octane (Acros Organics, 99+% extra dry), toluene (Acros Organics, 99.85% extra dry over molecular sieve), and cyclohexane (Acros Organics, 99.5% extra dry over molecular sieve). Nile Red (Roth), cyanine 3 NHS ester (Cy3, Lumiprobe), cyanine 5 NHS ester (Cy5, Lumiprobe), and PBI (AstaTech) were dissolved in toluene. Dye solutions were diluted in toluene, and QD solutions were diluted in octane, cyclohexane, or toluene before combining them in a mixture of QDs (≈10^–5^ mg/mL) and dye molecules (0.1–50 μM) in toluene. The final concentrations of dye molecules are given in Table S1. Subsequently, 100 μL of these solutions was spin-coated onto a cover glass (Thorlabs, 170 ± 5 μm thickness and 25 mm diameter) at 150 rps for 1 min. The samples were then placed in a home-built sample holder filled with a nitrogen atmosphere to preclude water and oxygen during the measurements.

Single-particle measurements were performed on a home-built uPL setup resembling an inverted epifluorescence microscope equipped with a 405 nm pulsed laser (PicoQuant, 10 MHz repetition rate, <50 ps pulse width, <100 W/cm^2^), which is focused with an oil immersion objective (1/*e*^2^ = 1 μm, 1.3 NA) onto the sample. The sample is mounted on XYZ translational stages (SmarAct, <1 nm resolution). The emitted light is collected by the same objective and passed through a dichroic mirror as well as a long-pass filter (both 450 nm cut-on wavelengths) to remove reflected excitation light. The collected and filtered light is sent to a monochromator coupled to an EMCCD (Princeton Instruments, one frame per second) to record the spectrum. Alternatively, to record PL intensity time traces, time-resolved PL traces, and photon–photon correlations, the collected and filtered light is sent to a modified HBT experiment consisting of a 50:50 beam splitter, two avalanche photodiodes (Excelitas, 250 ps time resolution), a time-correlated single-photon counting module (PicoQuant, HydraHarp), and a band-pass filter (Thorlabs, 510 nm, 42 nm bandwidth) in one arm and a long-pass filter (Thorlabs, 550 nm cut-on for Nile Red, Cy3, and PBI, 600 nm cut-on for Cy5) in the other arm. To record spectrally selective wide-field images of donor and acceptor PL, excitation light is focused onto the back focal plane of the objective with an additional lens, and collected light is additionally passed through a band-pass filter (Thorlabs, 510 nm, 42 nm bandwidth) to image green QD emission or through a long-pass filter (Thorlabs, 550 nm cut-on for Nile Red, Cy3, 600 nm cut-on for Cy5) to image dye emission and sent to an EMCCD (Princeton Instruments, 0.5 frame per second).

Data analysis was performed in Python 3.9.0 using NumPy 1.2.3, SciPy 1.6.2, pycorrelate 0.3, and spe2py 1.0.0a. The original file formats (.spe for EMCCD, .ptu for HBT experiment) were used directly. Intensity time traces and donor–acceptor correlation map were obtained by binning the photon arrival times at a binwidth of 10 ms and applying a median filter (kernel size = 11; except for Nile Red and bare QDs). Donor–acceptor correlation maps are histograms of the intensity traces obtained from the two arms of the HBT experiment. Normalized second-order correlation functions were constructed from photon arrival times using the pcorrelate function from pycorrelate using a binwidth of roughly 2 ns. Time-resolved PL traces were obtained by generating a histogram of the delays of the photons. The excited-state lifetime was defined as the time when the intensity decayed to 1/*e* of its initial value.

## Data Availability

All data supporting the findings in this study is available through Zenodo (doi:10.5281/zenodo.10869226).

## Supporting Information Available

Additional details on methods, sample characterization, ensemble time-resolved PL, single-dot experiments with additional donor–acceptor pairs and at cryogenic temperature (PDF)
