# Supplementary material for: Quantifying Förster Resonance Energy Transfer from Single Perovskite Quantum Dots to Organic Dyes
Source: ACS Nano. 2024 Mar 28;18(14):9997–10007. doi: 10.1021/acsnano.3c11359 (PMC11008358; doi:10.1021/acsnano.3c11359)
Supplement: Supplementary file 1 — nn3c11359_si_001.pdf [file nn3c11359_si_001.pdf]

Supporting Information for

Quantifying Förster Resonance Energy Transfer from Single Perovskite Quantum Dots to Organic Dyes

Leon G. Feld,[1,2,3] Simon C. Boehme,[1,2] Viktoriia Morad,[1,2] Yesim Sahin,[1,2,3] Christoph J. Kaul,[1,2] Dmitry N. Dirin,[1,2,3] Gabriele Rainò[1,2,3,] and Maksym V. Kovalenko[1,2,3,*]*

[1] Institute of Inorganic Chemistry, Department of Chemistry and Applied Biosciences, ETH Zürich, CH-8093 Zürich, Switzerland

[2] Laboratory for Thin Films and Photovoltaics, Empa – Swiss Federal Laboratories for Materials Science and Technology, CH-8600 Dübendorf, Switzerland

[3] National Centre of Competence in Research (NCCR) Catalysis, ETH Zürich, CH-8093 Zürich, Switzerland

Emails: mvkovalenko@ethz.ch, rainog@ethz.ch

Contents:

1 Synthesis and Characterization of QDs , Figure S1,S2 p. 1

2 Single-particle spectroscopy, Table S1 p. 4

3 Data analysis, Figure S3 p. 6

4 Spectral overlap and Förster Radius, Table S2 p. 8

5 Supporting Figures S4-S24 p. 9

1 Synthesis and Characterization of QDs

1.1 Synthesis

$PbBr_2$-trioctylphosphine oxide (TOPO) stock solution (0.067M). $PbBr_2$ (734 mg, 2.00 mmol, 99.999 % trace metals basis, Sigma Aldrich, stored in GB) and TOPO (4296 mg, 11.11 mmol, TOPO, 99 %, Stream, stored in GB) were dissolved in 25 ml n-octane (for synthesis ≥99%, Carl Roth) at 120 ◦C on a hotplate in air.

$ZnCl_2$-TOPO stock solution (0.067M). $ZnCl_2$ (273 mg, 2.04 mmol) and TOPO (4296 mg, 11.11 mmol) were dissolved in n-octane (25 ml) at 120 ◦C on a hotplate in air.

Cs-diisooctylphosphinic acid (DOPA) stock solution (0.02M). Cs_2CO_3 (97.8 mg, 0.30 mmol, 99.9% trace metals basis, Sigma Aldrich) and DOPA (1 ml, 3.15 mmol, technical ≈ 90%, Sigma Aldrich) were dissolved in 2 ml n-octane (for synthesis ≥99%, Carl Roth) at 120 ◦C on a hotplate in air. The reaction mixture is allowed to cool to room temperature, then diluted with 27 mL of n-hexane (suitable for HPLC ≥ 97.0%, Sigma Aldrich, stored over molecular sieves).

Ligand 2-octyl-1-dodecyl phosphoethanolamine (C_8C_{12}-PEA) was synthesized according to ref 1.

$CsPbBr_3$ QD synthesis. $PbBr_2$-TOPO (260 μL) was diluted with n-hexane (1 ml) and stirred on a stirring plate. CsDOPA (300 μL) was swiftly injected. QDs were allowed to grow for 60 seconds, and then 2 mg of C_8C_{12}-PEA in 20 μL of mesitylene was injected to stop the QDs' growth. For purification, QDs were precipitated with 1 eq. of EtOAc:ACN (2v:1v) mixture; the precipitate was collected and redispersed in n-hexane. The purification procedure was repeated twice and in the final step the NCs were redispersed in n-octane.

$CsPb(Br/Cl)_3$ QD synthesis. $PbBr_2$-TOPO (260 μL) and $ZnCl_2$-TOPO (100 μL) was diluted with n-hexane (1 ml) and stirred on a stirring plate. CsDOPA (300 μL) was swiftly injected. QDs were allowed to grow for 5 minutes, and then 2 mg of C_8C_{12}-PEA in 20 μL of mesitylene was injected to stop the QDs' growth. The purification procedure followed that of $CsPbBr_3$ sample above.

1.2 TEM characterization

TEM images were collected using the Hitachi HT7700 microscope, equipped with a tungsten/LaB_6 emitter and a double gap objective lens system, operated at 100 kV. TEM images were processed using the software ImageJ.

1.3 Ensemble optical characterization

The UV-Vis measurements were conducted with a V670 spectrometer from Jasco, equipped with a photomultiplier tube (PMT) and a Peltier-cooled PbS detector in transmission mode. The transmission spectra were corrected for a dark-counts spectrum and referenced to a baseline spectrum of the employed solvent.

The PL spectra were recorded with the Fluorolog iHR 320 Horiba Jobin Yvon spectrometer from Horiba Scientific fitted with a PMT detector. The PL emission was determined by finding the emission wavelength $\lambda_{emission}$ with maximum PL intensity.

Time-resolved PL measurements were performed with a FluoTime 300 spectrometer from PicoQuant equipped with a TimeHarp 260 PICO counting TCSPC unit and a 355 nm pulsed PicoQuant laser. Samples were prepared by spin-coating or drop-casting of solutions containing QDs (~0.1 mg/ml) and organic dyes (concentrations indicated in figure legends). Donor decay traces were recorded at the PL peak center using an emission monochromator.

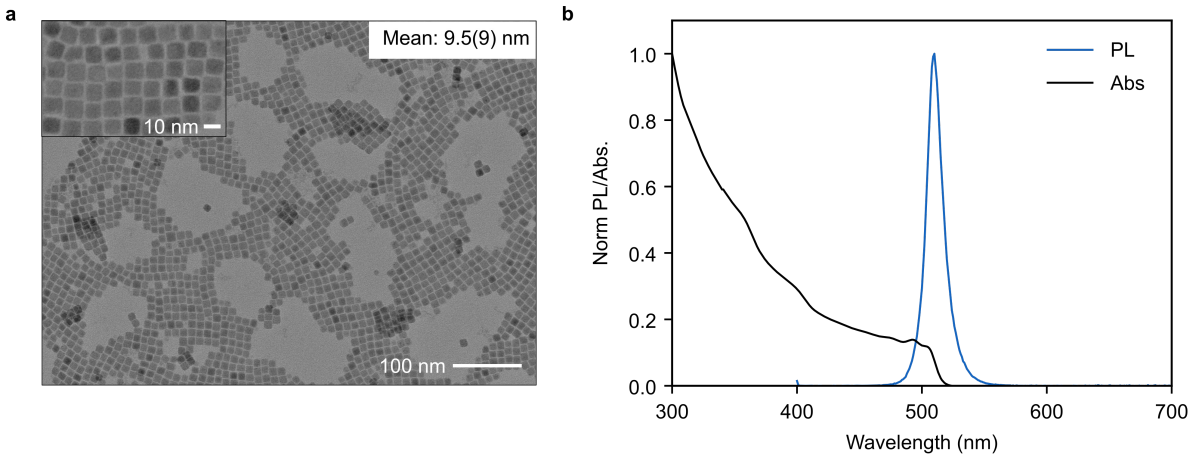

Figure S1: Ensemble characterization of the C_8C_{12}-PEA -capped $CsPbBr_3$ QDs. (a) TEM images of the sample with 9.5(9) nm edge lengths. (b) Normalized ensemble photoluminescence and absorption.

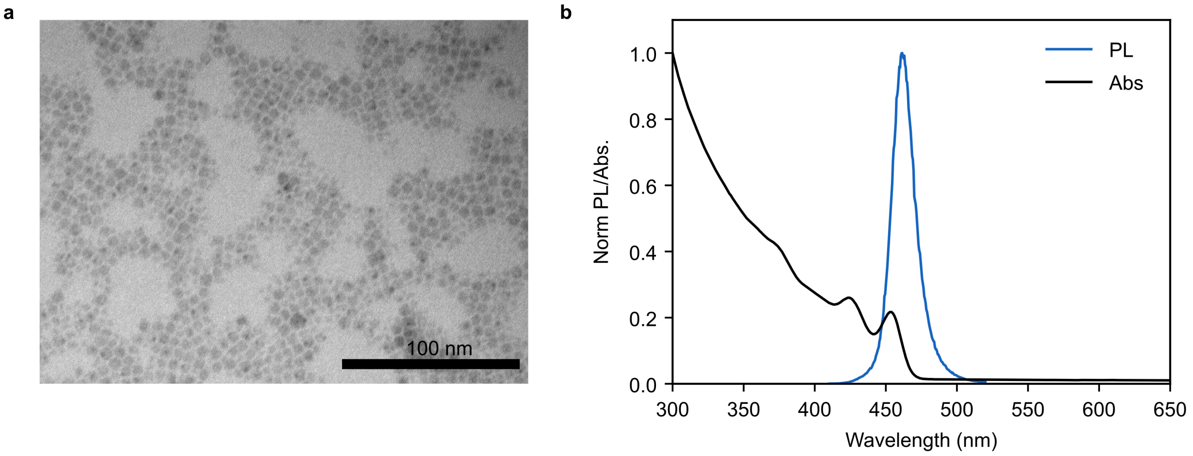

Figure S2: Ensemble characterization of the C_8C_{12}-PEA -capped $CsPb(Br/Cl)_3$ QDs. (a) TEM images of the sample with 5.3(6) nm edge lengths. (b) Normalized ensemble photoluminescence and absorption.

2 Single-particle Spectroscopy

2.1 Sample Preparation

The following steps were performed in a glovebox that is kept under a nitrogen atmosphere, employing dry and filtered octane (Acros Organics, 99+% extra dry), toluene (Acros Organics, 99.85% extra dry over molecular sieve) and cyclohexane (Acros Organics, 99.5% extra dry over molecular sieve). Nile Red (Roth), Cyanine 3 NHS ester (Cy3, Lumiprobe), Cyanine 5 NHS ester (Cy5, Lumiprobe) and N,N'-Bis(2,6-diisopropylphenyl)-1,6,7,12-tetraphenoxy-3,4,9,10-perylenetetracarboxylic Diimide (PBI, AstaTech) were dissolved in toluene. Dye solutions were diluted in toluene and QD solutions were diluted in octane, cyclohexane or toluene before combining them in a mixture of QDs (≈10^{-5} mg/ml) and dye molecules (0.1-50 µM) in toluene. The final concentrations of dye molecules are listed in Table S1. Subsequently, 100 µL of these solutions were spin-coated onto a cover glass (Thorlabs, 170 ± 5 µm thickness, and 25 mm diameter) at 150 rps for one minute. The samples were then placed in a home-built sample holder filled with nitrogen atmosphere to preclude water and oxygen during the measurements.

Table S1. Dye concentrations in QD-dye mixtures used for single-dot samples prior to spin-coating on clean glass coverslips at 150 rps for 60 seconds.

Dye	Concentration in spin-coated solution (in µM)
**Cyanine 3 NHS ester**	0.65
	2.17
	9.45
**Cyanine 5 NHS ester**	3.40
	34.05
**Nile Red**	0.144
	1.44
**PBI dye**	17.8

2.2 Optical Measurements

Single-particle measurements were performed on a home-built uPL setup resembling an inverted epifluorescence microscope equipped with a 405 nm pulsed laser (PicoQuant, 10 MHz repetition rate, <50 ps pulse width, <100 W/cm^2) which is focussed with an oil immersion objective (1/e^2 = 1 µm, 1.3 NA) onto the sample. The sample is mounted on XYZ translational stages (SmarAct). The emitted light is collected by the same objective and passed through a dichroic mirror as well as a long-pass filter (both 450 nm cut-on wavelengths) to remove reflected excitation light. The collected and filtered light is sent to a monochromator coupled to an EMCCD (Princeton Instruments, one frame per second) to record the spectrum. Alternatively, to record PL intensity time traces, time-resolved PL traces and

photon-photon correlations, the collected and filtered light is sent to a modified Hanbury-Brown and Twiss (HBT) experiment consisting of a 50:50 beam splitter, two avalanche photodiodes (Excelitas, 250 ps time resolution), a time-correlated single-photon counting module (PicoQuant, HydraHarp) as well as a band-pass filter (Thorlabs, 510 nm, 42 nm bandwidth) in one arm and a long-pass filter (Thorlabs, 550 nm cut-on for Nile Red, Cy3 and PBI, 600 nm cut-on for Cy5) in the other arm. To record spectrally selective widefield images of donor and acceptor PL, excitation light is focused onto the back focal plane of the objective with an additional lense and collected light is additionally passed through a band-pass filter (Thorlabs, 510 nm, 42 nm bandwidth) to image green QD emission or through a long-pass filter (Thorlabs, 550 nm cut-on for Nile Red, Cy3, 600 nm cut-on for Cy5) to image dye emission and sent to an EMCCD (Princeton Instruments, 0.5 frames per second).

3 Data analysis

The data analysis was performed in Python 3.9.0 using NumPy 1.2.3, SciPy 1.6.2, pycorrelate 0.3, and spe2py 1.0.0a. The original file formats (.spe for EMCCD, .ptu for HBT experiment) were used directly.

3.1 Intensity time traces and donor-acceptor correlation map

Denoised intensity time traces were obtained by binning the photon arrival times at a binwidth of 10 ms and applying a median filter (kernel size = 11; except for Nile Red and bare QDs). Donor-acceptor correlation maps are histograms of the intensity traces obtained from the two arms of the HBT experiment.

3.3 Second-order photon-photon correlation

Normalized second-order correlation functions were constructed from photon arrival times using the pcorrelate function from pycorrelate using a binwidth of roughly 2 ns.

3.4 Excited-state lifetime from time-resolved PL and FRET efficiency

Time-resolved PL traces were obtained by generating a histogram of the delays of the photons. The excited-state lifetime was defined as the time when the intensity has decayed to 1/e of its initial value.

The FRET efficiency can be obtained from the donor lifetime in presence of molecules $\tau(n)$ and in absence of molecules τ_{ref} which is obtained by measuring a reference sample:

$$E(n) = 1 - \frac{\tau(n)}{\tau_{ref}}$$

To determine sample-averaged single-particle efficiencies, we first determined τ_{ref} as the average lifetime across single QDs from a reference sample without dye. We could then compute the single-particle FRET efficiencies for individual QDs from samples with dye. Note that these values exhibit large variations, including negative values, due to large QD-to-QD variations in lifetime – even in absence of dye.

3.5 Minimum QD-center-to-dye distance from TEM

TEM images were collected for samples with closely packed QDs and denoised using a bandpass filter. We assume that the distance from the QD center to the center of the space between QDs (see yellow lines below) is a lower bound approximation for the minimum distance from the QD center to dye molecules. Using ImageJ, profiles along multiple QDs were extracted (an example is shown below). Peaks along these profiles correspond to the centers of the spaces between the QDs and the peak-to-peak distances correspond to twice the minimum center-to-dye distance. A mean peak-to-

peak distance of 11.58 nm with a standard deviation of 0.83 nm and a 95% confidence interval of 0.22 nm was determined.

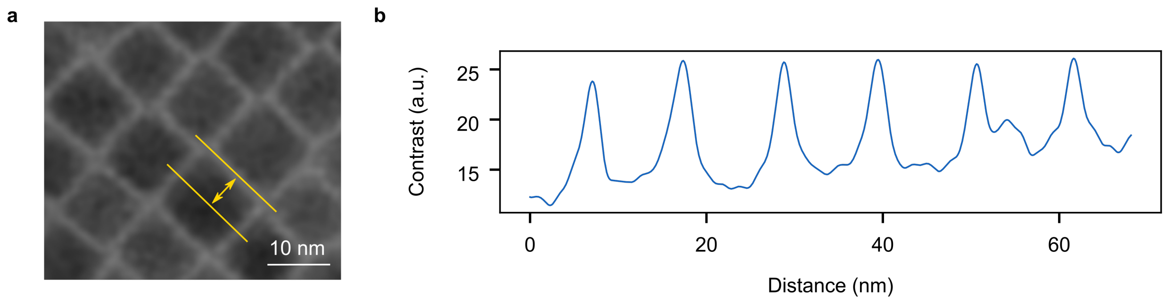

Figure S3: TEM-based analysis of the smallest QD center-to-dye distance in absence of dye molecules. (a) Yellow lines and arrows indicate the minimum center-to-dye distance which corresponds to the distance from the QD center to the center of the space between the QDs. (b) Profile along multiple QDs obtained from a TEM image. Peaks in the image contrast correspond to the space between QDs.

4 Spectral overlap and Förster radius

4.1 Spectral overlap

The FRET efficiency is proportional to the spectral overlap $J(\lambda)$ of donor emission and acceptor absorption. We used the a|e tool (https://www.fluortools.com/software/ae-uv-vis-ir-spectral-software) to calculate the spectral overlap between our QD emission and dye absorption in toluene. Using peak extinction coefficients of 150000 L $mol^{-1}cm^{-1}$ for Cyanine 3 and 250000 L $mol^{-1}cm^{-1}$ for Cyanine 5 and ensemble PL and absorbance measurements in toluene, we obtain the spectral overlaps listed in Table S2.

Table S2: Spectral overlaps for different QD-dye pairs in this work.

**Donor-acceptor pair**	**Overlap integral** [$nm^4M^{-1}cm^{-1}$]
**$CsPbBr_3$-Cy3**	3.2×10^{15}
**$CsPbBr_3$-Cy5**	4.4×10^{14}
**$CsPb(Br/Cl)_3$-Cy3**	3.9×10^{14}

4.2 Förster radius

The Förster radius can be obtained within a point-dipole approximation as

$$R_0 = 0.221 \left(\frac{\kappa^2\ \Phi_D J(\lambda)}{n^4}\right)^{\frac{1}{6}},$$

with κ^2 being an orientation factor (2/3 for freely rotating donor and acceptors), Φ_D the donor photoluminescence quantum yield (we used 0.9), $J(\lambda)$ the spectral overlap of donor emission and acceptor absorption, and n is the refractive index. Estimating the refractive index is very difficult due to the limited knowledge of the local environment of the dye. We roughly estimate the refractive index of the medium as

$$n = \frac{2}{3}\ 2.4 + \frac{1}{3}\ 1.37 = 2.06\ ,$$

where we chose 2.4 as the refractive index of the QD core and 1.37 (refractive index of hexane) for the ligand shell. Using these values, we obtain Förster radius of 4.59 nm for the FRET pair of $CsPbBr_3$ QDs and Cy3.

5 Supporting Figures

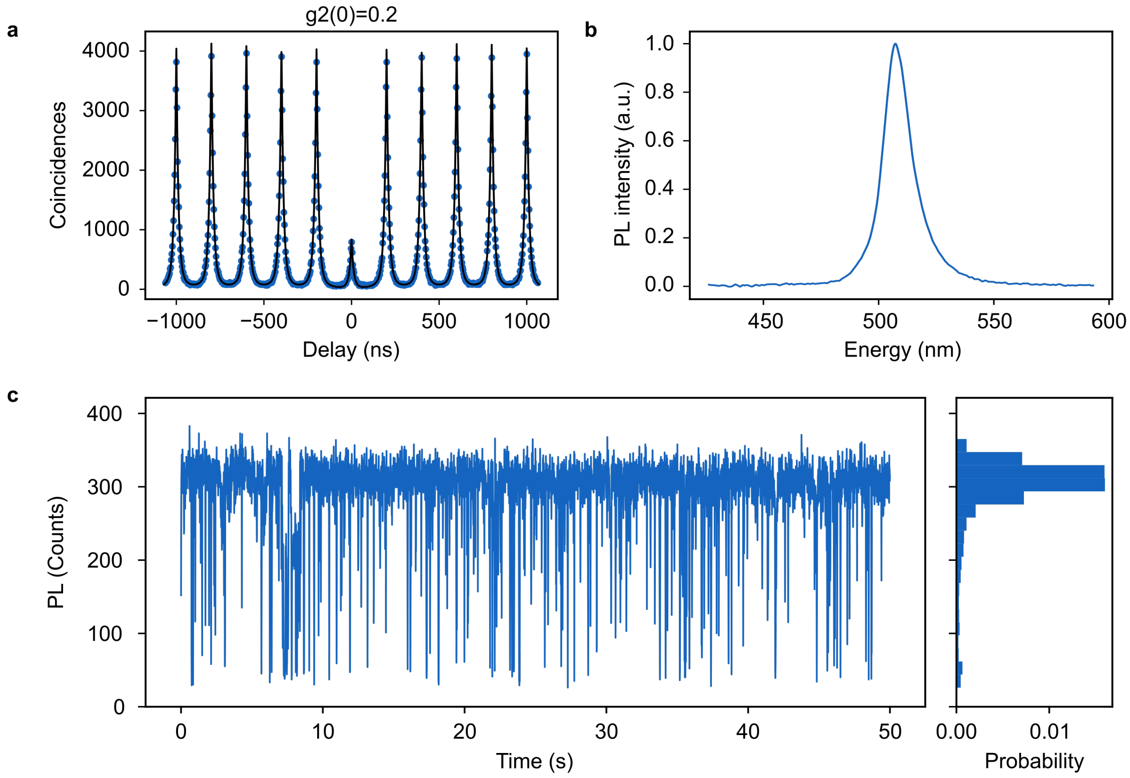

Figure S4: Single-particle characterization of C_8C_{12}-PEA capped $CsPbBr_3$ QDs. Representative second-order photon-photon correlation function (a), PL spectrum (b) and intensity trace with 10 ms bin width (c).

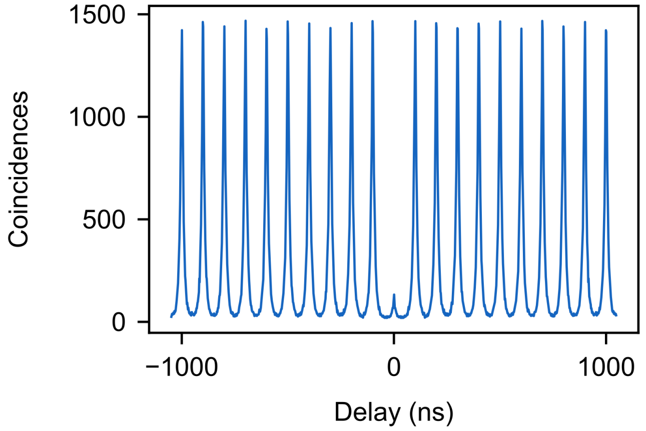

Figure S5: Second-order intensity correlation function $g^2(\tau)$ of the emission of a single $CsPbBr_3$ QD in a film of Cy3. The green QD emission was selectively probed with a bandpass filter (510 nm).

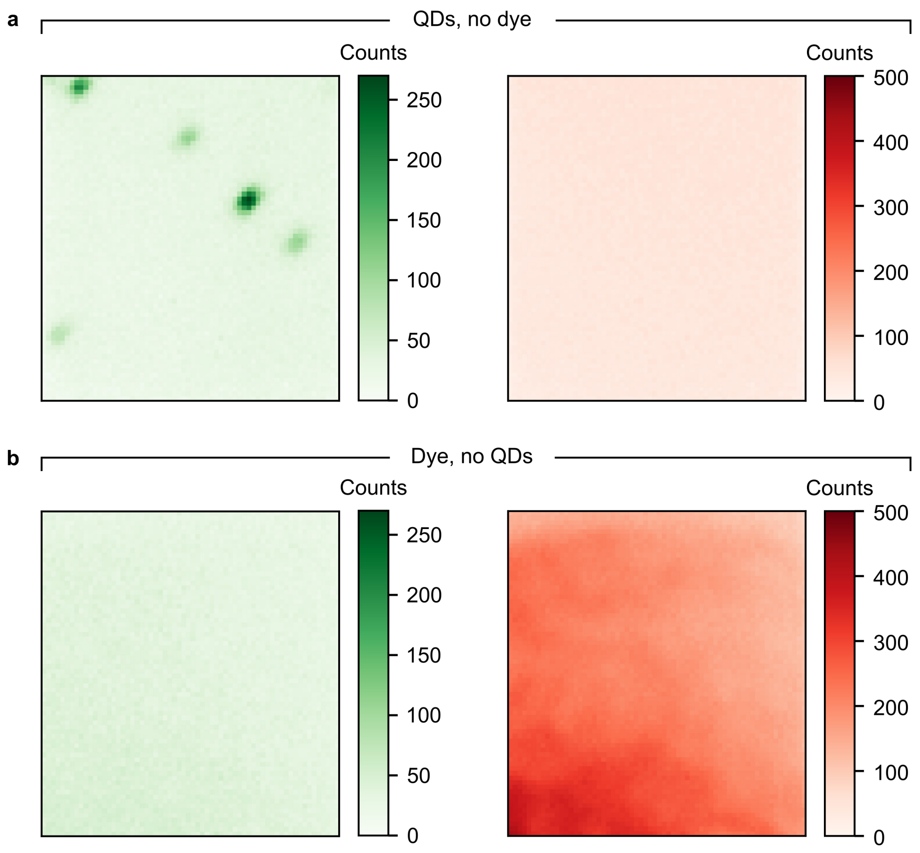

Figure S6: Control experiments for widefield images in Figure 2b of the main text. (a) Widefield images in the green (left) and red channel (right) of a sample with QDs but without Cy3. Green emission from QD can be observed, whereas red emission is not observable due to the lack of dyes. (b) Widefield images in the green (left) and red channel (right) without QDs and with Cy3 feature a continuous signal in the red channel due to direct excitation of Cy3 at 405 nm but no emission in the green channel due to the lack of QDs.

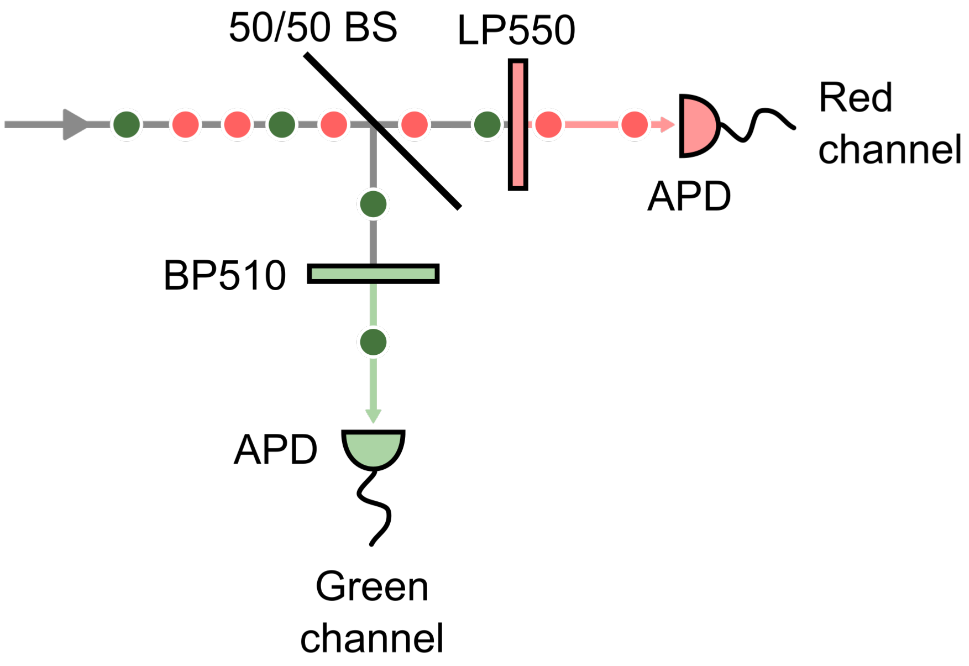

Figure S7: Modified Hanbury-Brown and Twiss setup to correlate green donor and red acceptor emission at single-photon (green and red dots) resolution. BS is a 50/50 beam splitter, LP550 is a long-pass filter with a cut-on wavelength of 550 nm, BP510 is a band-pass filter with a central wavelength of 510 nm and a bandwidth of 42 nm, APD is an avalanche photodiode.

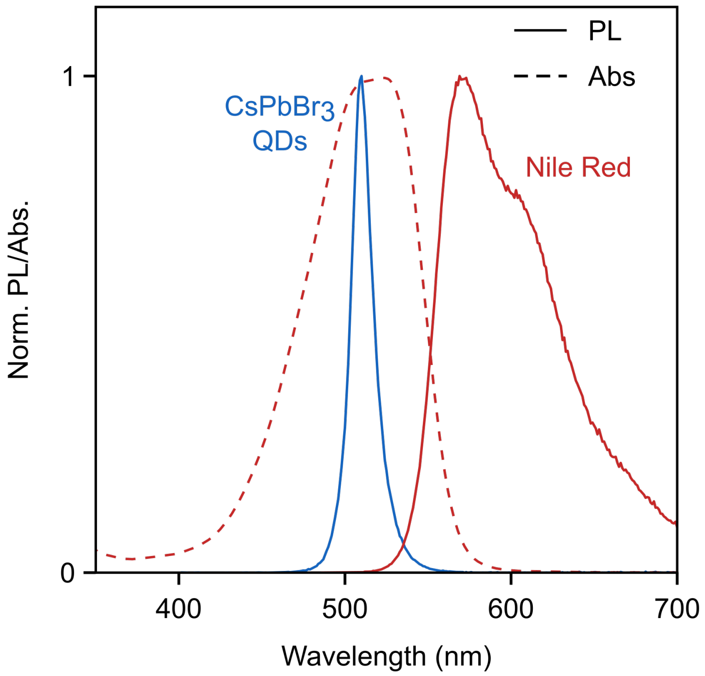

Figure S8: PL and absorption of Nile Red in toluene compared to PL of QDs in toluene.

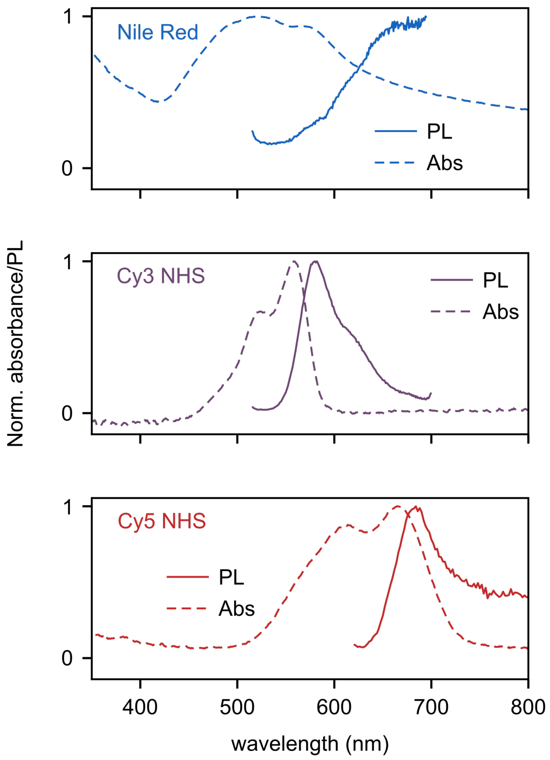

Figure S9: PL and absorption of Nile Red, Cy3 and Cy5 in films. PL is hardly observable for Nile Red films.

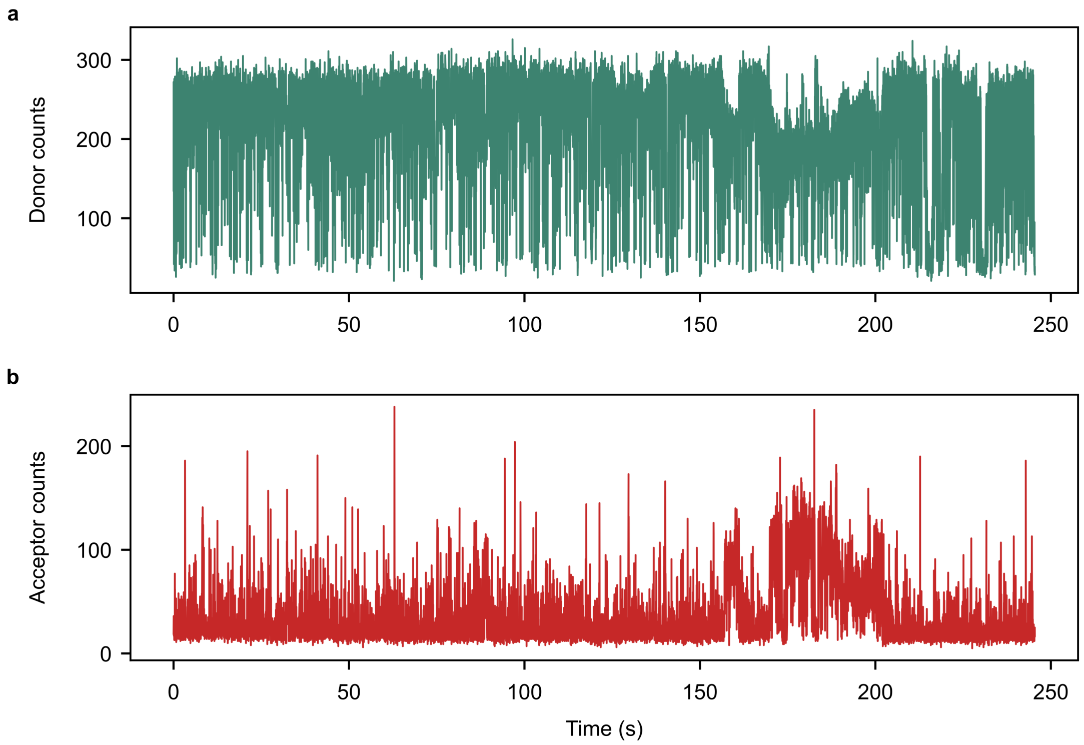

Figure S10: Intensity time traces of samples with single QDs surrounded by Nile Red recorded in the green (a) and red (b) channels. Temporal donor-acceptor correlations can be observed featuring an anticorrelated fast flickering. Note that median filtering was not applied to the Nile Red traces. Note that the vertical axis in (a) does not include zero counts.

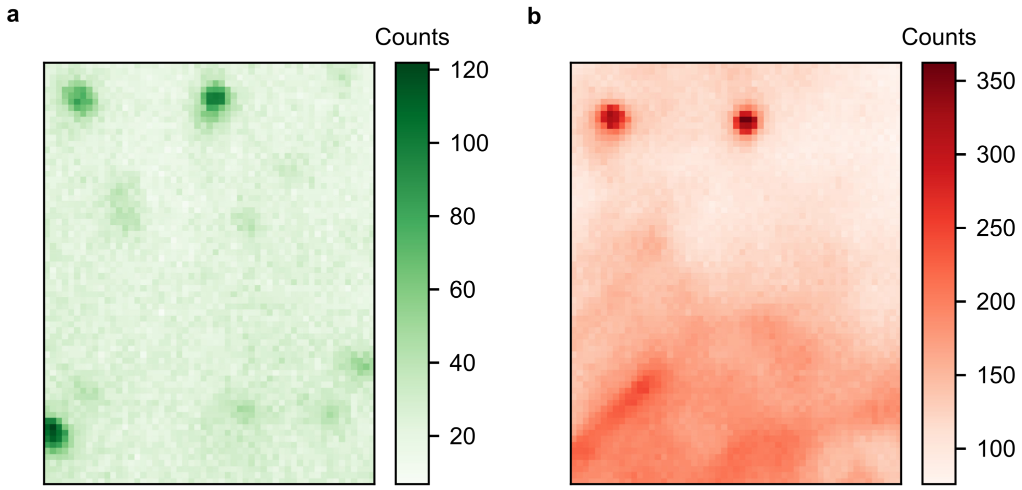

Figure S11: Spatial correlation of QD and Nile Red emission. (a) The image in the green channel shows bright spots where QDs are located. (b) In the red channel, bright spots correlate well with those in the green channel; the continuous background originates from the direct excitation of Nile Red.

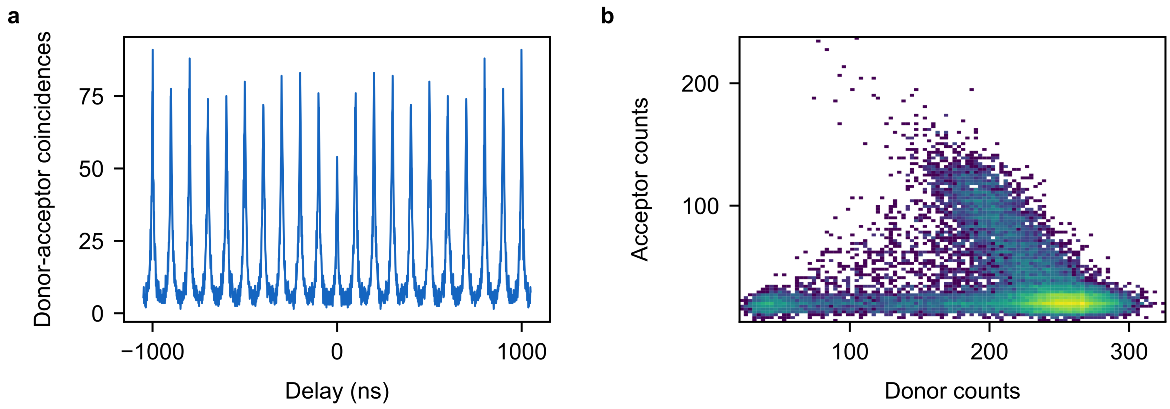

Figure S12: Second-order intensity correlation function $g^2(\tau)$ (a) and correlation map (b) for the emission from QDs (green channel) and Nile Red (red channel). Note that axes in (b) do not include zero counts.

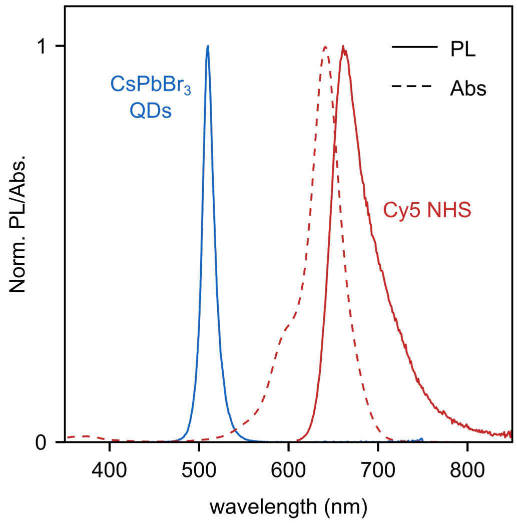

Figure S13: PL and absorption of Cyanine 5 NHS ester (Cy5) in toluene compared to PL of QDs in toluene, with negligible spectral overlap between QD emission and dye absorption.

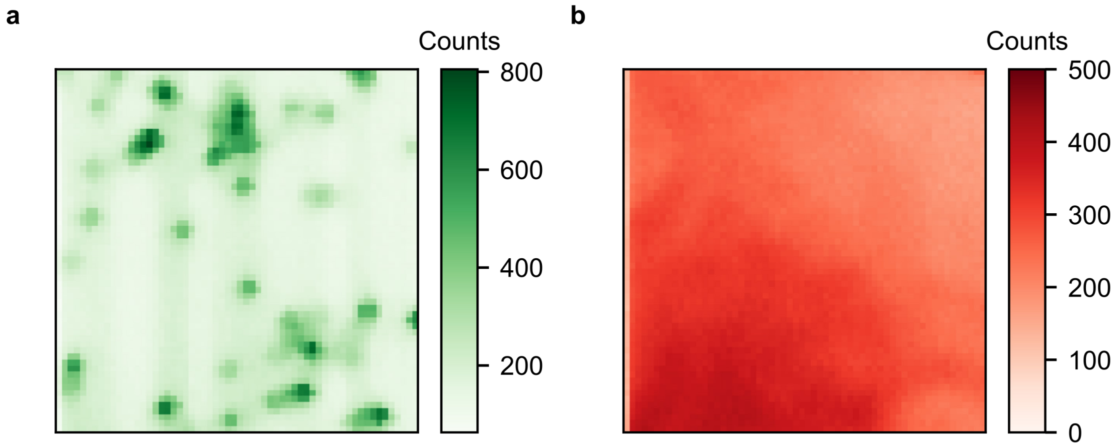

Figure S14: Widefield images of samples with QDs and Cy5. (a) Image of the green QD emission show bright spots from the QD emission. (b) Selectively imaging the red emission of Cy5 yields no bright spots and instead shows spatially homogeneous emission due to direct excitation of Cy5.

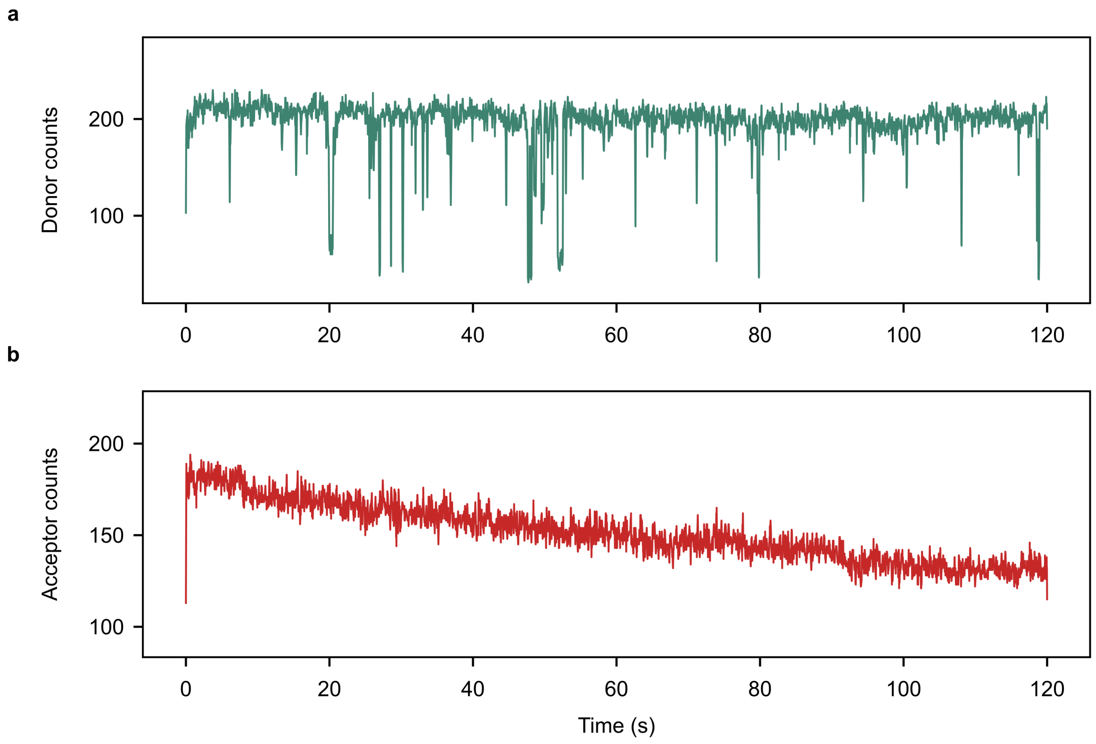

Figure S15: Intensity time traces of samples with single QDs and Cy5 recorded in the green (a) and red (b) channel, with an absence of temporal correlations between QD and dye emission. Note that vertical axes do not include zero counts.

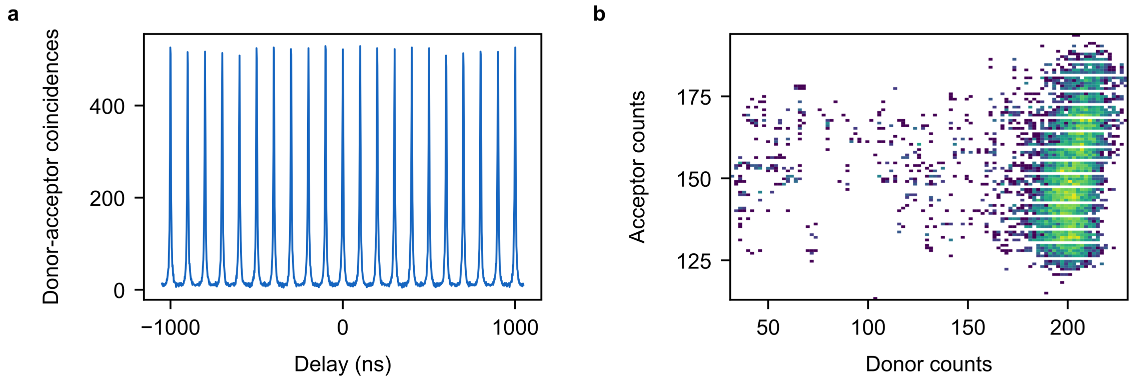

Figure S16: Second-order intensity correlation function $g^2(\tau)$ (a) and correlation map (b) for the emission from QDs (green channel) and Cy5 (red channel). No intensity or photon correlations are observable. Note that axes in (b) do not include zero counts.

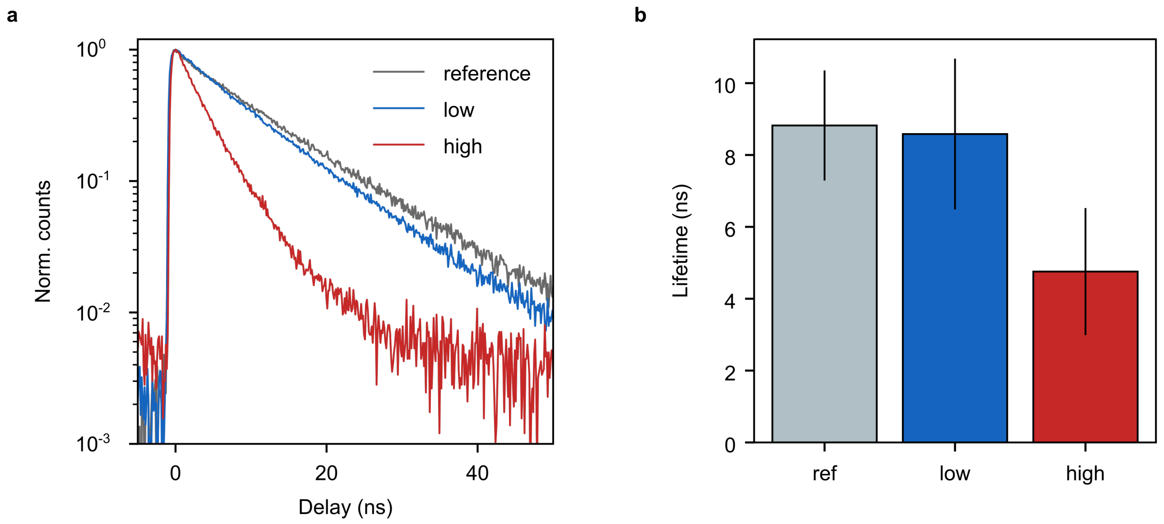

Figure S17: Time-resolved PL of QDs in the presence of Cy5. (a) Representative time-resolved PL traces for different concentrations of Cy5. (b) Histogram of the obtained lifetimes from the reference and at different concentrations of Cy5. Error bars indicate standard deviations.

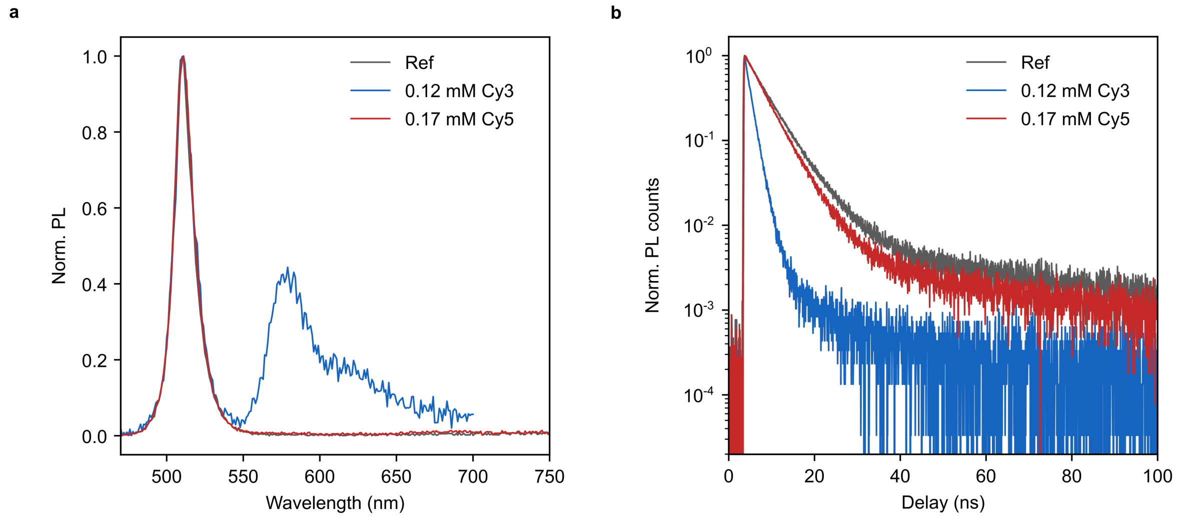

Figure S18: Ensemble PL measurement of drop-casted films of $CsPbBr_3$ QDs with similar concentrations of Cyanine 3 and Cyanine 5 NHS ester. (a) Steady-state PL spectrum normalized to the peak arising from QD emission. Concentrations in the legend correspond to the dye concentration in the solutions before drop-casting. (b) TRPL traces of the QD emission around 510 nm display shorter QD (donor) lifetimes for Cy3 than for Cy5. Extracted 1/e decay times indicate FRET efficiencies of 71% and 8%, respectively. Concentrations in the legend correspond to the dye concentration in the solutions before drop-casting.

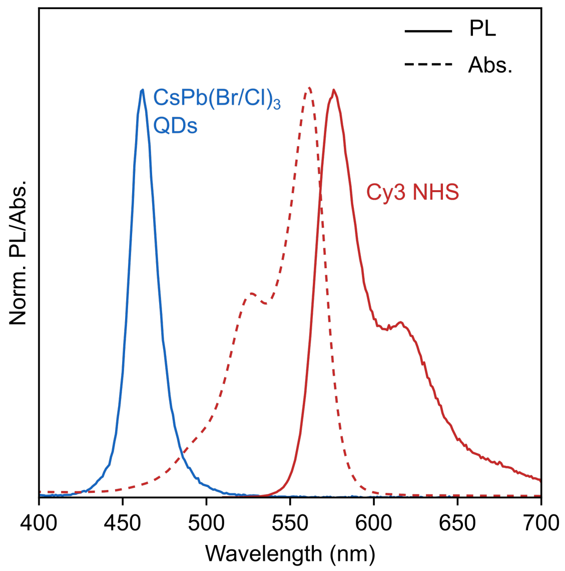

Figure S19: Ensemble PL spectra of $CsPb(Br/Cl)_3$ QDs (blue solid line) and Cyanine 3 NHS ester (red solid line) and absorption spectrum of Cyanine 3 NHS ester (red dashed line).

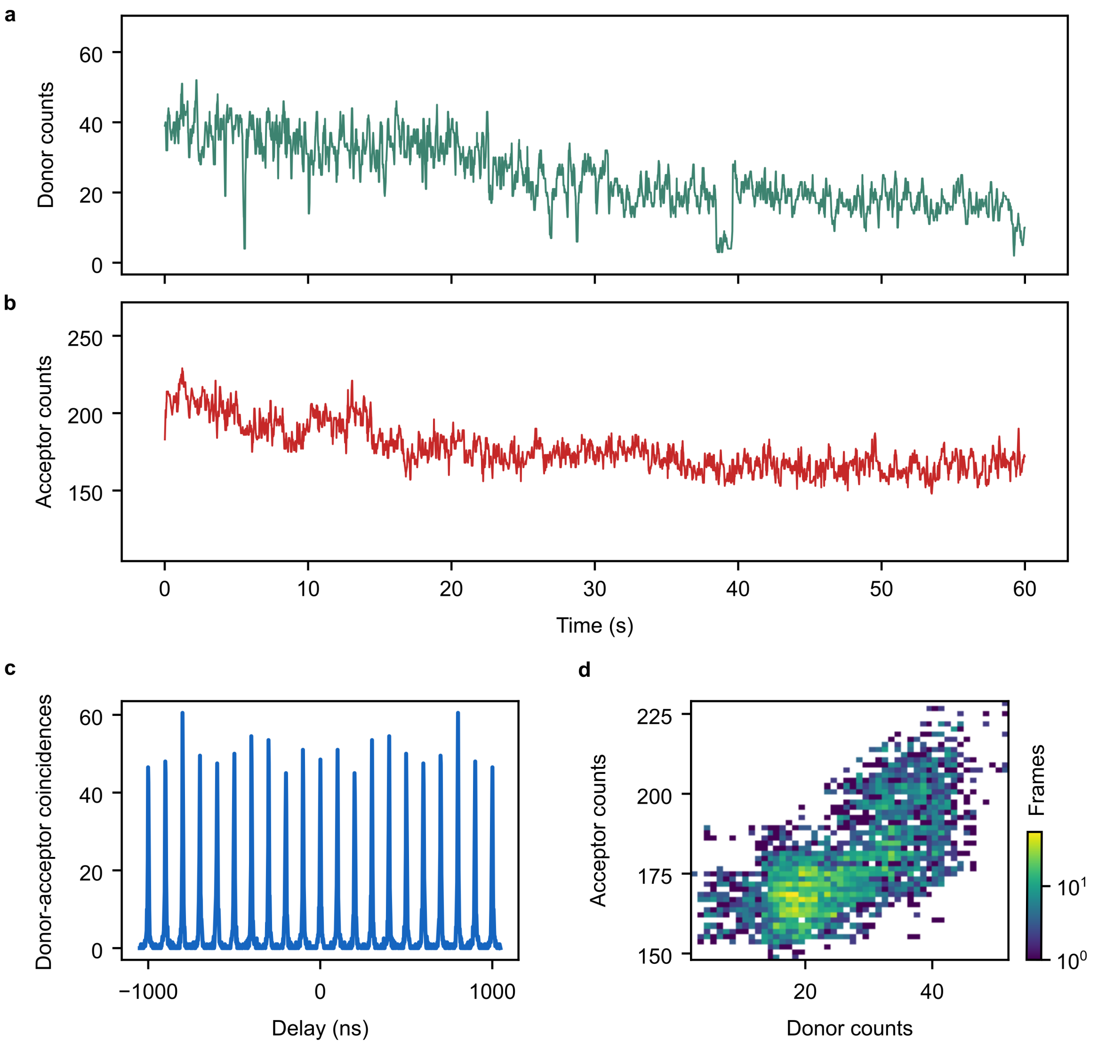

Figure S20: Representative single-particle PL measurement of a $CsPb(Br/Cl)_3$ QD in presence of a large concentration of Cyanine 3 NHS ester molecules exhibiting no signs of energy transfer. (a,b) Intensity time trace of green donor (a) and red acceptor (b) emission displaying no pronounced correlation or anti-correlation intensity jumps. Note that the vertical axis in (b) does not include zero counts. (c) Intensity correlation map of the red donor and green acceptor emission. (d) Second-order photon-photon correlation of donor and acceptor photons. Note that axes do not include zero counts.

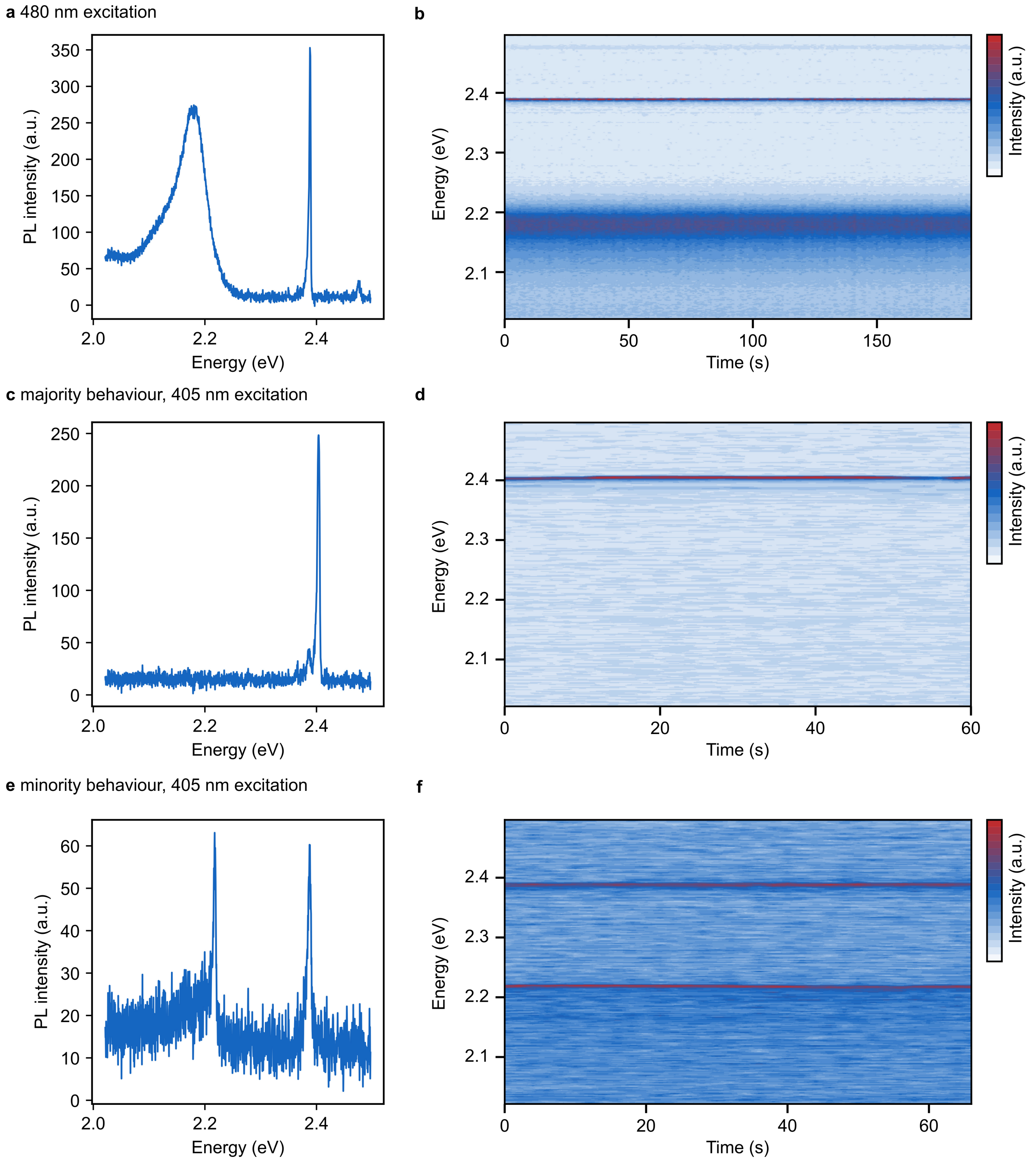

Figure S21: Single-particle PL spectroscopy of $CsPbBr_3$ QDs in a film of Cyanine 3 NHS ester at cryogenic temperatures (8.1 K). (a,b) Representative PL spectrum (a) and spectra series (b) recorded under 480 nm excitation displaying a sharp signal from a single $CsPbBr_3$ QD and a broad signal from a large number of directly excited Cyanine 3 molecules. (c,d) Representative PL spectrum (c) and spectra series (d) recorded under 405 nm excitation displaying a sharp signal from a single $CsPbBr_3$ QD. (e,f) Uncommon PL spectrum € and spectra series (f) recorded under 405 nm excitation displaying a sharp signal from a single $CsPbBr_3$ QD and a sharp signal from a Cyanine 3 molecule excited through the QD.

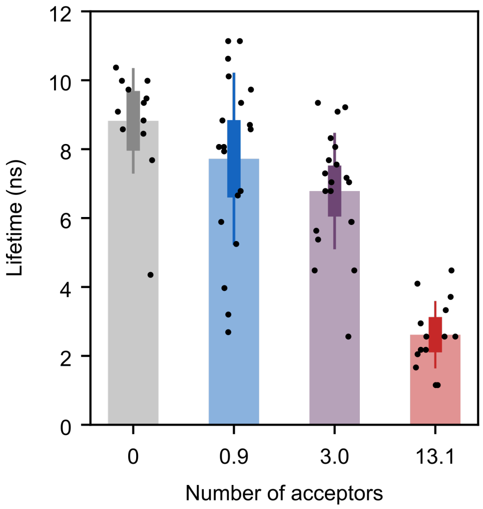

Figure S22: Statistics of donor lifetime at increasing concentrations of Cyanine 3 molecules. Datapoints correspond to individual QDs. Numbers in the x-axis to the estimated mean number of acceptor molecules. Bars indicate the sample-averaged lifetime, while thick error bars correspond to 95% confidence intervals and thin error bars indicate standard deviations.

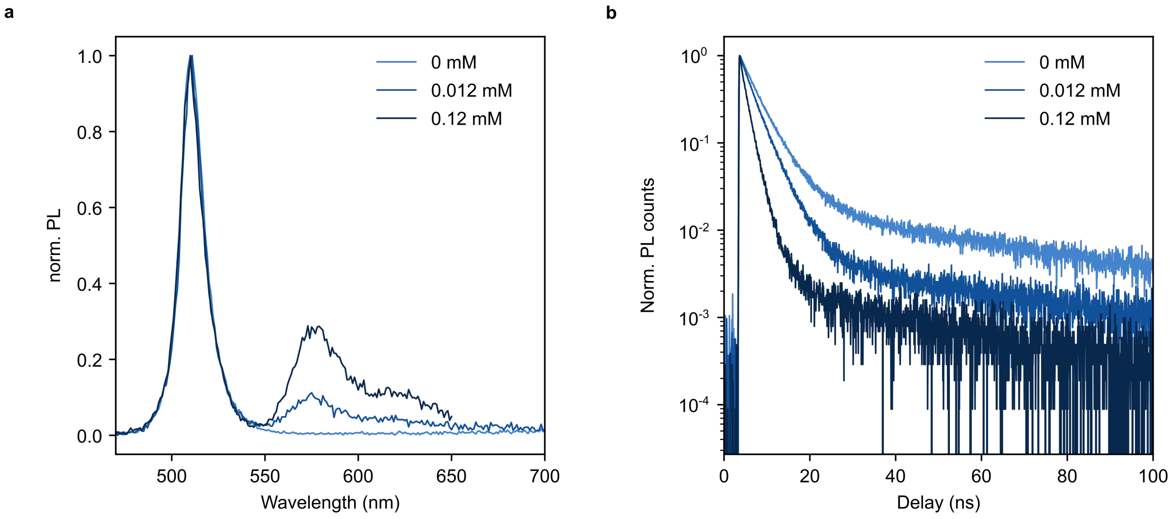

Figure S23: Ensemble PL measurement of spin-coated films of $CsPbBr_3$ QDs with increasing concentrations of Cyanine 3 NHS ester. (a) Steady-state PL spectrum normalized to the peak arising from QD emission. Concentrations in the legend correspond to the dye concentration in the solutions before spin-coating. (b) TRPL traces of the QD emission around 510 nm display shortening of the QD (donor) lifetime with increasing dye concentration. Extracted 1/e decay times indicate FRET efficiencies of 32% and 77%, respectively. Concentrations in the legend correspond to the dye concentration in the solutions before spin-coating.

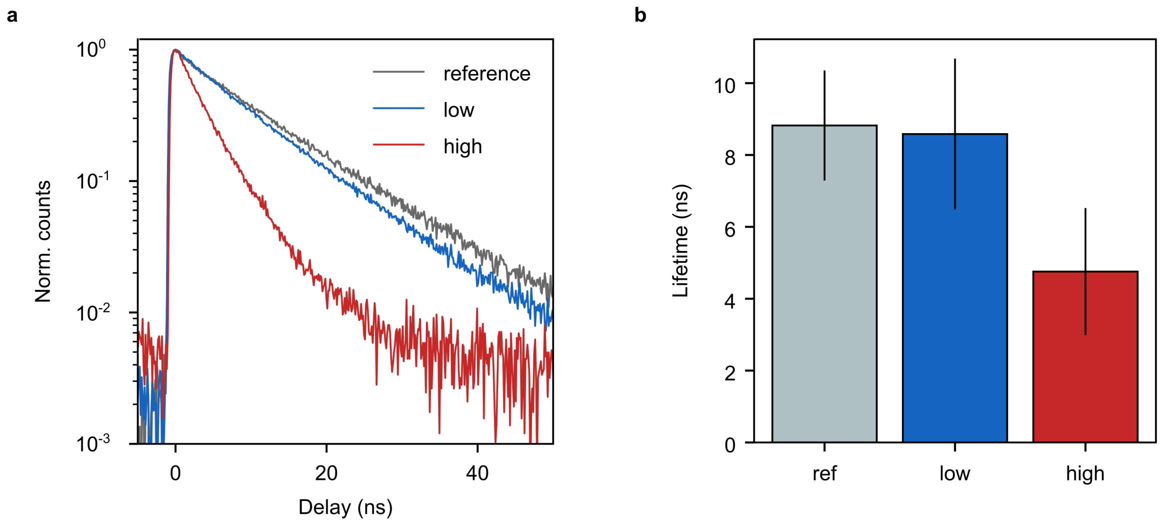

Figure S24: Time-resolved PL of QDs in the presence of Nile Red. (a) Representative time-resolved donor PL traces for different concentrations of Nile Red. (b) Histogram of the obtained lifetimes from the reference and different concentrations of Nile. At the highest concentration, the quenched lifetime corresponds to a transfer efficiency of 46(22)%. Error bars correspond to standard deviations.

References

(1) Morad, V.; Stelmakh, A.; Svyrydenko, M.; Feld, L. G.; Boehme, S. C.; Aebli, M.; Affolter, J.; Kaul, C. J.; Schrenker, N. J.; Bals, S.; et al. Designer Phospholipid Capping Ligands for Soft Metal Halide Nanocrystals. *Nature* **2024**, *626*, 542-548. DOI: 10.1038/s41586-023-06932-6
